# Quantitative and Discriminative Evaluation of Contents of Phenolic and Flavonoid and Antioxidant Competence for Chinese Honeys from Different Botanical Origins

**DOI:** 10.3390/molecules23051110

**Published:** 2018-05-08

**Authors:** Shi Shen, Jingbo Wang, Qin Zhuo, Xi Chen, Tingting Liu, Shuang-Qing Zhang

**Affiliations:** National Institute for Nutrition and Health, Chinese Center for Disease Control and Prevention, 29 Nanwei Rd, Beijing 100050, China; wangjb@ninh.chinacdc.cn (J.W.); zhuoqin@ninh.chinacdc.cn (Q.Z.); chenxi@ninh.chinacdc.cn (X.C.); Liutt@ninh.chinacdc.cn (T.L.)

**Keywords:** UPLC-MS/MS, antioxidant competence, phenolic, flavonoid, chemometrics, monofloral honey

## Abstract

Phenolics and flavonoids in honey are considered as the main phytonutrients which not only act as natural antioxidants, but can also be used as floral markers for honey identification. In this study, the chemical profiles of phenolics and flavonoids, antioxidant competences including total phenolic content, DPPH and ABTS assays and discrimination using chemometric analysis of various Chinese monofloral honeys from six botanical origins (acacia, *Vitex*, linden, rapeseed, *Astragalus* and *Codonopsis*) were examined. A reproducible and sensitive ultra-performance liquid chromatography-tandem mass spectrometry (UPLC-MS/MS) method was optimized and validated for the simultaneous determination of 38 phenolics, flavonoids and abscisic acid in honey. Formononetin, ononin, calycosin and calycosin-7-*O*-β-d-glucoside were identified and quantified in honeys for the first time. Principal component analysis (PCA) showed obvious differences among the honey samples in three-dimensional space accounting for 72.63% of the total variance. Hierarchical cluster analysis (HCA) also revealed that the botanical origins of honey samples correlated with their phenolic and flavonoid contents. Partial least squares-discriminant analysis (PLS-DA) classification was performed to derive a model with high prediction ability. Orthogonal partial least squares-discriminant analysis (OPLS-DA) model was employed to identify markers specific to a particular honey type. The results indicated that Chinese honeys contained various and discriminative phenolics and flavonoids, as well as antioxidant competence from different botanical origins, which was an alternative approach to honey identification and nutritional evaluation.

## 1. Introduction

Honey is usually defined as “the natural sweet substance produced by *Apis mellifera* bees from the nectar of plants or from secretions of living parts of plants or excretions of plant-sucking insects or the living parts of plants, which the bees collect, transform by combining with specific substance of their own, deposit, dehydrate, store, and leave in honeycombs to ripen and mature” [1]. Honey consists essentially of different sugars, predominantly fructose and glucose as well as enzymes and other phytochemicals such as volatile compounds, amino acids, phenolics and flavonoids, which are affected by the floral origin, the contamination with propolis, and external factors such as climate, geographical origin and processing conditions [2,3,4,5,6]. Honey has been widely used not only as a sweetener, but also used for both medical and nutritional purposes, which include antimicrobial [7] anti-inflammatory [8] and anti-oxidant properties [9,10]. Usually, honeys are classified as monofloral and polyfloral, and monofloral honey generally has better taste and higher economic value than polyfloral honey, therefore, the identification and nutritional evaluation of monofloral honey deserves great attention.

The construction of chemical profiles composed of the characteristic compounds for certain monofloral honeys might be considered as an alternative approach to honey identification, e.g., Oroian et al. [11,12,13] reported that the physicochemical properties such as moisture content, conductivity, 5-HMF, fructose and glucose, and volatile compounds could be used for the classification of Romanian honeys in different botanical and geographical origins. Compared to other floral markers, the phenolics and flavonoids are more often used, exhibit a wide range of biological effects and act as natural antioxidants [14,15], e.g., hesperetin is specific for citrus honey [5] and gallic acid could be a useful marker for manuka honey [16]. In 2016, it was reported that the production of Chinese honey reached 481,400 tons and accounted for 12.32% of the total exported amounts of honey worldwide [17,18]. A couple of analysis of phenolic and flavonoid compounds from honey in China using high-performance liquid chromatography (HPLC) with diode-array detection (DAD), electrode coulometric detection (ECD) detector and tandem mass spectrometry were reported [19,20]. Owing to the presence of conjugated double and aromatic bonds in the structure, flavonoids and phenolics exhibit the maximum absorbance in the vicinity of 280 nm and 360 nm in the UV region. ECD based on the measurements of the current resulting from oxidation/reduction reaction of the analyte at suitable electrode coulometric array detection provides selectivity and sensitivity for the analysis of flavonoids. HPLC-DAD-MS/MS was developed for the determination of only 12 phenolic and flavonoid compounds in chaste and rape honeys and kaempferol, morin and ferulic acid were used as floral markers to distinguish them [19]. HPLC–ECD measured the contents of only 13 phenolic acids in Chinese honey samples (jujube, longan and chaste) and a chemometric analysis was constructed with phenolic acids as variables for the identification of their floral origin [20]. Nevertheless, the identification of the compounds with similar UV–Vis spectra, and the oversensitivity of the ECD method to the temperature, speed, oxygen and impurity in a mobile phase are very challenging in the analysis of flavonoids and phenolics from the complexity of the honey matrix. The mass spectrometry equipped with electrospray ionization (ESI) shows high sensitivity and has been widely employed for structural confirmation and quantitative analysis based on molecular mass. Compared to HPLC analysis, ultra-performance liquid chromatography (UPLC) has high resolution, speed and sensitivity, especially UPLC-MS/MS method can provide higher accuracy and precision and has been widely applied in foodomics and natural product analysis. In China, besides traditional monofloral honeys studied before, there are other monofloral honeys collected from the nectar of traditional Chinese medicines (TCMs), such as *Astragalus* and *Codonopsis*, which are claimed to have various nutritional values and consumer preferences due to specific compounds of TCM, for instance, formononetin, ononin, calycosin and calycosin-7-*O*-β-d-glucoside, however there is rare report on the phytochemical constituents of specific Chinese honeys from TCMs. Therefore, it is necessary to establish a comprehensive analysis of phenolic and flavonoid profiles from Chinese monofloral honeys and the possible correlations between more compounds and floral origins to cater to the market demand.

This study aims to develop a comprehensive determination method for contents of phenolics and flavonoids as many as possible using solid phase extraction (SPE) and ultra-performance liquid chromatography-tandem mass spectrometry (UPLC-MS/MS), nutritionally evaluate the antioxidant competences according to total phenolic content, DPPH and ABTS radical scavenging activities, discriminate different floral origins using chemometric analysis for six types of monofloral honeys (acacia, *Videx*, linden, rapeseed, *Astragalus* and *Codonopsis* honeys) in China.

## 2. Results and Discussion

### 2.1. Optimization and Method Validation of UPLC-MS/MS Method

The UPLC-MS/MS method was developed and validated for simultaneous quantification of 38 phenolics, flavonoids and (±)-abscisic acid from honey samples. Chromatograms of the standard solution are presented in Figure S1. Several experiments were performed to evaluate different mobile phases consisting of methanol or acetonitrile as an organic phase and water as an aqueous phase, with different concentrations of formic acid (0.01%, 0.05% and 0.1%, *v*/*v*). The highest concentration of formic acid (0.1%, *v*/*v*) in water (mobile phase A) and acetonitrile (mobile phase B) provided overall better peak shape and degree of separation. In addition, the gradient was optimized in order to provide a good separation of the 38 compounds in 20 min. Compared to the BEH C18 column, a High Strength Silica (HSS) T3 column was able to provide a better adequate separation and delivered symmetrical peaks, e.g., chrysin, kaempferol, luteolin, morin and myricetin, because HSS T3 was ideally suited for the enhanced retention of polar compounds and metabolites by reversed-phase LC, and enabled analytes to more readily access the pore structure of the material, providing a balanced retention of polar and hydrophobic molecules without the need for ion-pair reagents. Other parameters such as column temperature, flow rate and injection volume were optimized to get a reliable separation. Testing temperatures from ambient temperature, 30 °C, 35 °C and 40 °C indicated that retention times decreased slightly with increasing column temperature and the best results were achieved at 35 °C resulting in narrow peaks and good separation between compounds. The flow rate was screened at levels of 0.2, 0.3 and 0.5 mL/min and 0.3 mL/min showed appropriate run time and good separation. The injection volume of 1.0 and 2.0 μL were evaluated and 1.0 μL provided symmetric and narrow peak shape.

Using the optimized conditions, the compounds were determined quantitatively in the MRM acquisition mode. Considering most of compounds eluted from 5–10 min centrally, segmented MS scan time according to the retention time of each compound was extraordinarily necessary to ensure efficient collection point numbers and the sensitivity of the method. Though structures of flavonoid compound were rather similar and some of them had the identical precursor and product ions, they could be differentiated by retention time from LC separation, e.g., morin (*t*_R_ = 8.29 min), quercetin (*t*_R_ = 8.85 min) and hesperetin (*t*_R_ = 10.02 min); Some compounds had the identical retention time but could be separated based on MS information, e.g., luteolin (*t*_R_ = 8.76 min) and calycosin (*t*_R_ = 8.77 min). Therefore, satisfactory separation performances were obtained from both LC and MS separation which ensured the MRM selectivity, as shown in Table 1 and Figure S1.

The validation results of the method are presented in Table 2. An external standard calibration for each analyte was made by diluting standard solutions using the mobile phase and the calibration linear ranges for all analytes were constructed by plotting the standard analytes peak area versus concentration, and the correlation coefficients were higher than 0.995 for all analytes. The limit of quantification (LOQ) was the lowest concentration at which an acceptable signal/noise ratio ≥ 10 could be achieved. The limit of detection (LOD) was the lowest concentration of compound that was not necessarily quantitative but was distinguishable from zero (signal/noise ratio ≥ 3). The LOD and LOQ values obtained in this study ranged from 0.01 to 28.49 μg L^−1^ and from 0.03 to 113.97 μg L^−1^, respectively. The intra-day precision was examined by injecting a standard solution of all analytes at medium concentration levels of calibration curves in six independent replicates on the same day. The inter-day precision was evaluated by the same analyst over three different days. Intra-day and inter-day precision expressed as relative standard deviations were achieved from 1.87% to 9.31% and 1.50% to 8.68%, respectively. Extraction recoveries were evaluated by testing a solution of *Vitex* honey (4 g in 20 mL of water acidified to pH 2) spiked with tested compounds (250 μg L^−1^ each) on the solid phase extraction. Each compound was determined using the formula:

Recovery% = [(C − C1)/C0] × 100%(1)

where C0 is the concentration of the compound in the initial standard solution, and C1 is the concentration of the spiked sample which was determined from calibration curves, and C is the calculated concentration. Excellent recoveries ranged from 56.07–106.66% for the analytes were obtained using an Oasis HLB cartridge. According to previous reports [21,22], using 10 mL of methanol as eluent, recoveries of flavonoids such as kaempferol, myricetin, quercetin, and luteolin were less than 20%. Considering some flavonoids have strong affinities for C18 sorbent, 30 mL of methanol was employed to ensure satisfactory recoveries.

### 2.2. Quantitative Determination of Phenolic and Flavonoids Compounds in Honey

Table 3 and Table 4 list the contents of 19 flavonoid aglycones, five flavonoid glycosides, 13 phenolic acids and (±)-abscisic acid from six types of 66 honey samples. Contents of 38 compounds for each sample are given in Tables S1–S4. High amounts of flavonoid aglycones and low amounts of glucosides maybe caused by bee saliva enzymolysis. Eight different phenolic acids (ferulic, isoferulic, syringic, 4-hydroxybenzoic, 3,4-dihyroxybenzoic, caffeic, *p*-coumaric and salicylic acid), seven flavonoids (chrysin, pinocembrin, apigenin, naringenin, luteolin, kaempferol and quercetrin) and (±)-abscisic acid were identified in all honey samples, in which 4-hydroxybenzoic acid (162.31–2255.48 ng g^−1^) and (±)-abscisic acid (65.09–746.47 ng g^−1^) were the most abundant compounds. Four flavonoids of formononetin, ononin, calycosin, and calycosin-7-*O*-β-d-glucoside were found for the first time in honey samples. 2-hydroxycinnamic acid, fisetin and morin were not identified in any sample.

For acacia honey, sinapic acid, genistein, (−)-epigallocatechin, baicalin, gaillic acid, chlorogenic acid, rutin and hesperidin were found as well. Compared to other honeys, the contents of 3,4-dihydroxybenzoic acid, gallic acid and *p*-coumaric acid were lower, (−)-epigallocatechin and hesperedin were higher. In *Vitex* honey, vitexin, gallic acid, quercetin, chlorogenic acid and rutin were also found. Only *Vitex* honey contained 3-hydroxybenzoic acid; calycosin, calycosin-7-*O*-β-d-glucoside and myricetin were not found in *Vitex* honey. The contents of chrysin, pinocembrin, apigenin, luteolin, kaempferol, vitex, 4-hydroxy benzoic acid, gallic acid, *p*-coumaric acid, caffeic acid and chlorogenic acid in *Vitex* honey were significantly higher than those in other five types of honeys. For linden honey, quercetin can be found in all samples. (−)-epigallocatechin and 3,4-dihydroxybenzoic acid were abundant, and sinapic acid, genistin, calycosin and calycosin-7-*O*-β-d-glucoside were not detected in linden honey. In rapeseed honey, sinapic acid, syringic acid, heseperetin, myricetin, gallic acid and isorhamnetin were found. Compared to other five types of honeys, higher contents of syringic acid (44.48–140.82 ng g^−1^) and sinapic acid (2.41–7.94 ng g^−1^), and lower contents of abscisic acid, chlorogenic acid, 4-hydroxybenzoic acid and 3,4-dihydroxybenzoic acid existed in rapeseed honey. Formononetin, genistein, calycosin, calycosin-7-*O*-β-d-glucoside, (−)-epigallocatechin and 3-hydroxybenzoic acid were not observed in rapeseed honey. Sinapic acid, hesperetin, quercetin, myricetin, ononin, formononetin, calycosin, calycosin-7-*O*-β-d-glucoside, gallic acid and isorhamnetin were detected in *Astragalus* and *Codonopsis* honeys. Specially, ononin, formononetin, calycosin and calycosin-7-*O*-β-d-glucoside were first reported and quantified in honeys, which were specific secondary metabolites from nectar plant of TCM and can be used for floral markers. (−)-Epigallocatechin, genistin and 3-hydroxybenzoic acid were not identified in *Astragalus* and *Codonopsis* honeys.

### 2.3. Antioxidant Activity

Honey is considered as a rich source of antioxidant activity mainly due to the presence of phenolic acids and flavonoids. The total phenolic content (TPC) ranged from 9.43 mg GAE/100 g in acacia honey to 26.78 mg GAE/100 g in *Codonopsis* honey. The DPPH radical scavenging activity (DPPH-RSA) of analyzed honey samples ranged from 7.11% in rapeseed honey to 51.42% in linden honey. The antioxidant content determined in terms of antioxidant equivalent ascorbic acid content (AEAC) values for DPPH radical scavenging activity, ranged from 3.09 mg AEAC/100 g in rapeseed honey to 24.02 mg AEAC/100 g in linden honey. The ABTS + radical scavenging activity (ABTS + -RSA) of analyzed various honey samples ranged from 59.10% in acacia honey to 86.46% in linden honey. The antioxidant content determined in terms of AEAC values for ABTS + radical scavenging activity, ranged from 27.37 mg AEAC/100 g in acacia honey to 38.05 mg AEAC/100 g in linden honey. The data are presented as mean ± standard deviation values in Table 5.

Dark honey possessed higher phenolic content and consequently higher antioxidant activity as compared to light colored honey [23]. The Pearson’s correlation values (*p* < 0.01) between TPC and DPPH-RSA (r = 0.798), TPC and DPPH-AEAC (r = 0.810), TPC and ABTS-RSA (r = 0.807), TPC and ABTS-AEAC (r = 0.816), indicated that phenolic content contributed to the radical scavenging activity of the analyzed honeys.

### 2.4. Chemometric Analysis

#### 2.4.1. Principal Component Analysis

**Figure 1:**
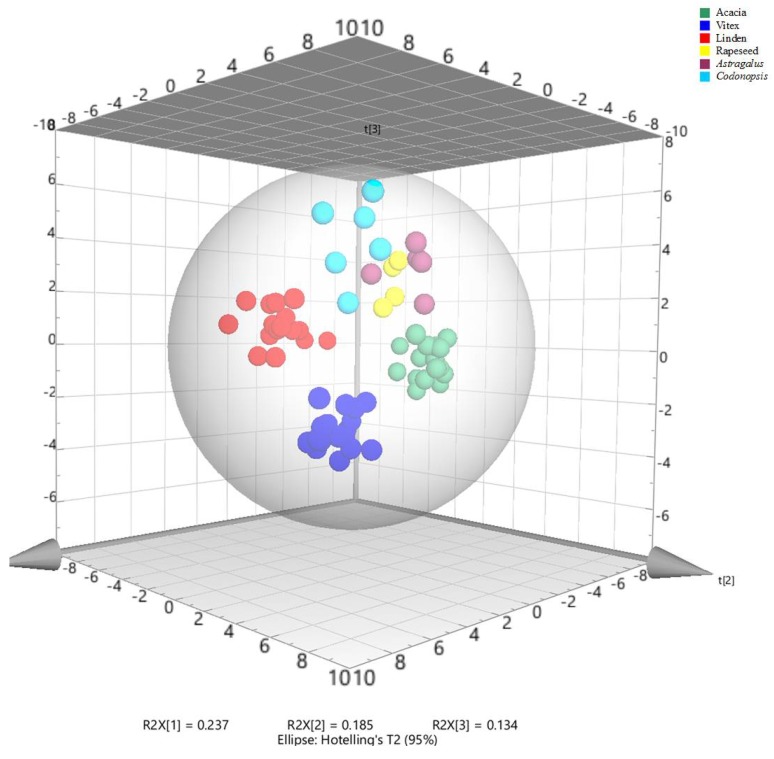
3D PCA scores plots of honeys.

Principal component analysis (PCA) was performed on the data for 40 variables (35 compounds, TPC, DPPH RSA, ABTS RSA, AEAC for DPPH and ABTS radical scavenging activities) in 66 honey samples (acacia, *Vitex*, linden, rapeseed, *Astragalus*, and *Codonopsis*) to investigate the distribution of honey samples from different botanical origins. The total six principle components can explain 72.63% of the total variance, the first principal component (PC1) represented 23.7% of the variance, and the next two principal components represented 18.5% and 13.4% of the variance, respectively. As shown in the PCA score plot (Figure 1), the samples studied were discriminated into six different groups which corresponded with their botanical origins. The samples of acacia honey were indicated by the negative axis of PC1. Linden honeys were mostly distributed in the positive axis of PC1 and negative of PC2. *Vitex* honeys were located in the positive axis of PC1 and negative of PC3. The samples of rapeseed, *Astragalus* and *Codonopsis* honey were closely located in the positive axis of PC3, *Codonopsis* honeys were indicated by the positive axis of PC1 and PC2, and *Astragalus* honeys were characterized by the positive axis of PC2, and rapeseed honeys were in the negative axis of PC1. PCA is an unsupervised technique, meaning that it shows the main structure in the data without considering a special direction or type of information. It was already clear in the PCA score plot that the six types of honeys were discriminated, and especially *Acacia*, *Vitex*, linden and *Codonopsis* honeys were obviously different.

#### 2.4.2. Hierarchical Cluster Analysis

**Figure 2:**
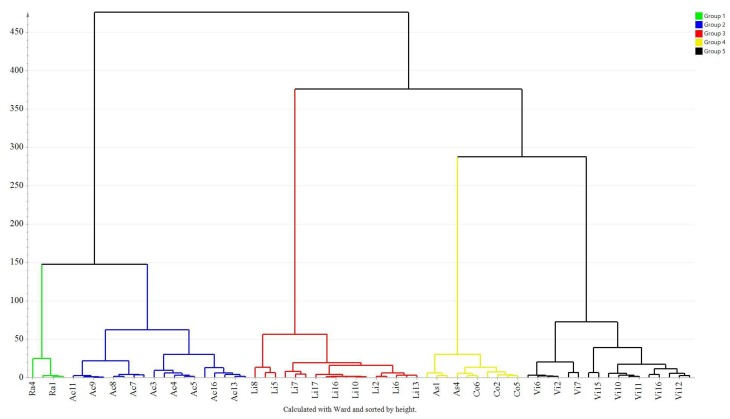
Dendrogram of HCA for honeys. Ra: rapeseed (*Brassica campestris* L.); Ac: Acacia (*Robinia pseudoacacia* L.); Li: linden (*Tiliaamurensis* Rupr.); As: Astragalus (*Astragalus membranaceus* (Fisch.) Bunge); Co: Codonoposis (*Codonopsis pilosula* (Franch.) Nannf.); Vi: vitex (*Vitex negundo* var. *heterophylla* Rehd.).

Hierarchical cluster analysis (HCA) was applied to the data including phenolic and flavonoid compounds as well as antioxidant competence studied to describe the overall nearness between honey samples. The Euclidean distance was used as a distance measure to calculate the sample similarities between the honey samples, and the parameters of the clustering algorithm and linkage rule were set as Ward’s and hierarchical values, respectively. Figure 2 shows the results of HCA as a dendrogram. The heights of the clusters are proportional to the Euclidean distance between the clusters. The algorithm has successfully grouped all the honey samples into five clusters, from left to right were rape (green), acacia (blue), linden (red), the mixture of *Astragalus* and *Codonopsis* (yellow) and *Vitex* (black) honey samples at a linkage level of 150. At a linkage level of 60, *Astragalus* and *Codonopsis* honey samples were separated. Those indicated that the clusters acacia honey, *Vitex* honey, linden honey and rapeseed honey were far in Euclidean distances, whereas the *Astragalus* honey and *Codonopsis* honey clusters were close. The results of HCA were consistent with those of PCA, indicating the difference of discrimination among clusters.

#### 2.4.3. Discriminant Analysis

The good discrimination from the analysis of the unsupervised pattern recognition prompted us to perform a partial least squares-discriminant analysis (PLS-DA) classification to construct a model with high prediction ability. All the samples were randomly separated into 55 known samples and 11 validating (predicting) samples as unknown belonging to the training set and test set respectively. The PLS-DA model built on the training set showed high discrimination, with R2X = 0.755, R2Y = 0.859, and Q2 = 0.727. The results of correct and ambiguous classifications obtained for the test set are displayed in Table 6. Each sample was classified by means of the probability of fitting the models of class membership indicative of its representativeness. As shown in Table 6, when the “probability of fitting the models of class membership” is larger than 0.5 (highlighted in bold), the object is considered correctly predicted. Also, all the commercial test samples for prediction fit the model space defined by the training set using Hotelling’s T2 range algorithm in 95% confidence interval. The honey samples declared to be acacia, *Vitex*, linden, *Astragalus* and *Codonopsis* honeys, respectively, were correctly classified evidently, however, the sample declared to be rapeseed slightly fit the probability because of less than 0.5 of the classification index.

**Figure 3:**
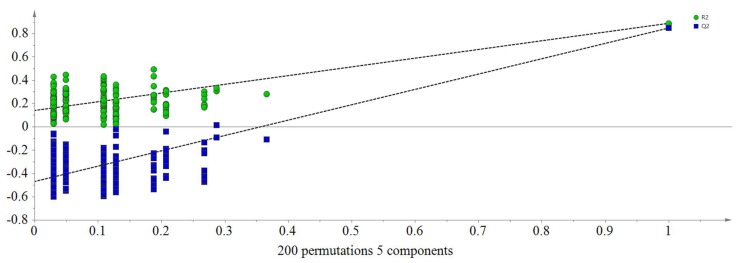
Permutation test (200 cycles, R2 = 0.888, Q2 = 0.847).

An orthogonal partial least squares-discriminant analysis (OPLS-DA) model was constructed by paired analysis of each honey and the rest, which provided the information of the correlations between specific markers and each particular honey type. Q^2^ (cum) parameters for samples from acacia, *Vitex*, linden, rapeseed, *Astragalus* and *Codonopsis* origins were 0.881, 0.899, 0.886, 0.720, 0.558 and 0.575, respectively, which showed acceptable predictability for honeys from different floral origins (Figures S2–S7). Cross validation was evaluated using a permutation test with 200 cycles, where R^2^ and Q^2^ were calculated as the goodness of fit and the predictive capability of the model, respectively. Generally, the larger values of slope for R2 and Q2 and the larger value of difference between two parameters represented an excellent model, as shown in Figure 3.

**Figure 4:**
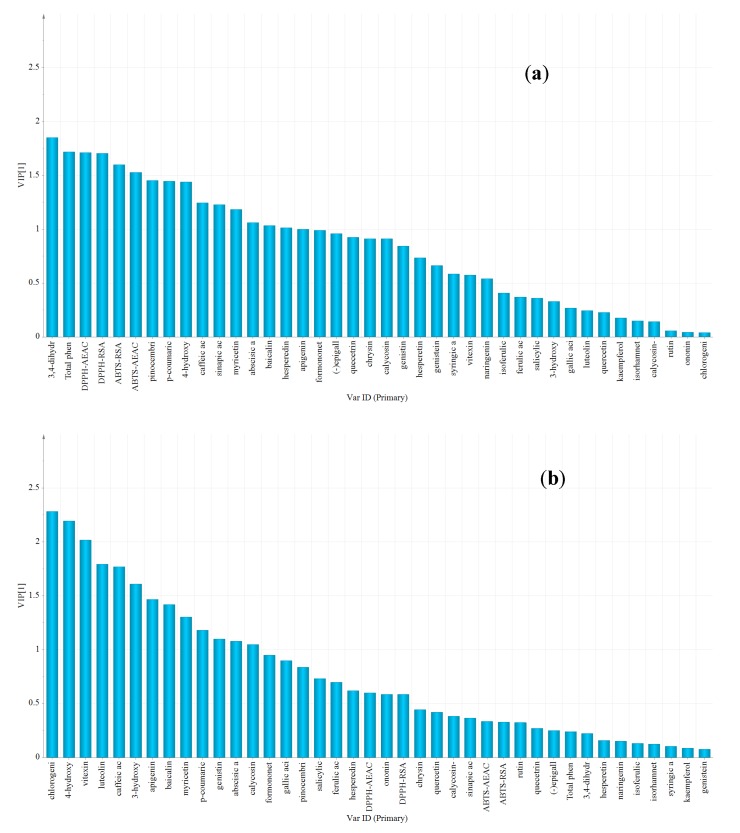

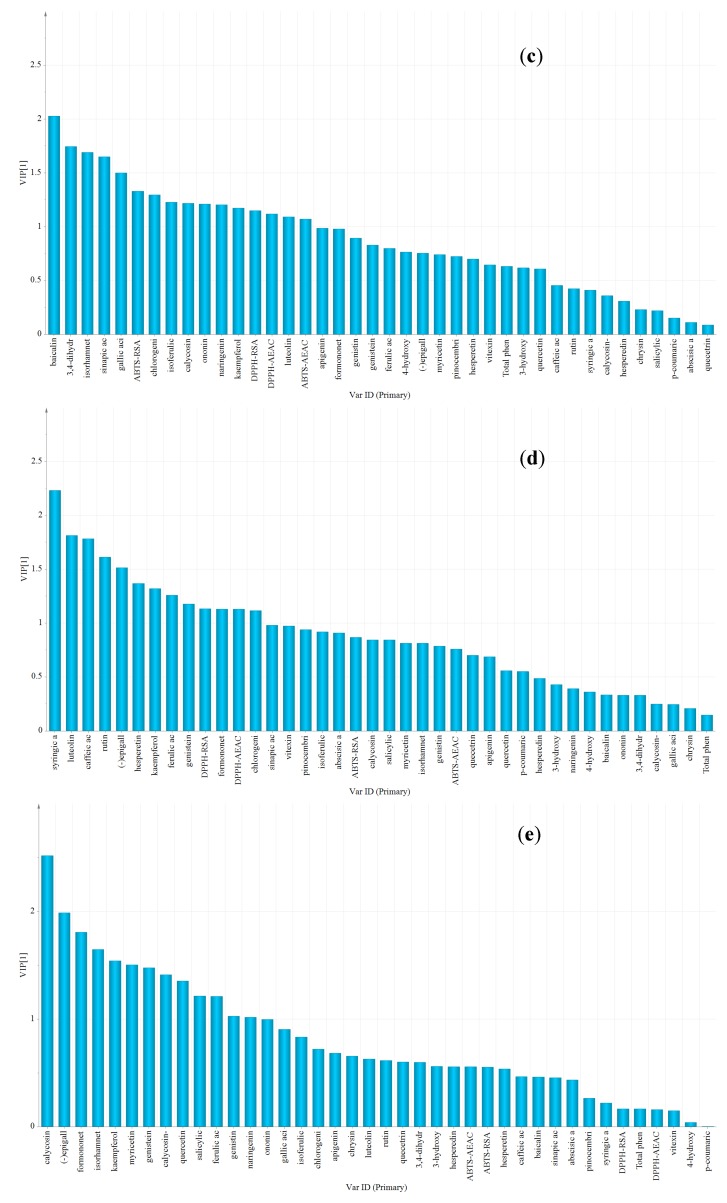

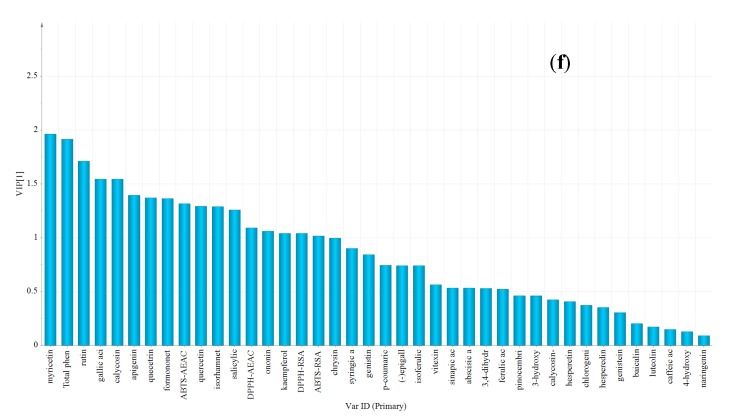
The VIP plot by OPLS-DA corresponding with discrimination between (**a**) Acacia versus rest; (**b**) Vitex versus rest; (**c**) Linden versus rest; (**d**) Rapeseed versus rest; (**e**) Astragalus versus rest and (**f**) Codonopsis versus rest.

The significance of variables was evaluated using the variable importance in the projection (VIP) method, and the specific variables were determined according to VIP value of more than 1.5 [24]. Overall, acacia honey was distinguished from other honeys because of lower contents of 3,4-dihydroxybenzoic acid, TPC, pinocembrin, *p*-coumaric acid, 4-hydroxybenzoic acid, and lower values of DPPH-RSA, DPPH-AEAC, ABTS-RSA and ABTS-AEAC antioxidant properties than other honeys (Figure 4a). *Vitex* honey was obviously identified from other honeys based on the highest contents in chlorogenic acid, 4-hydroxybenzoic acid, vitexin, luteolin, caffeic acid and 3-hydroxy-benzoic acid (Figure 4b). Linden honey samples were characterized by a higher content of baicalin and 3,4-dihyroxybenzoic acid, a lower content of isorhamnetin and gallic acid and no detection of sinapic acid (Figure 4c). Rapeseed honey samples showed high correlations with a higher content of syringic acid, and a lower content of luteolin, caffeic acid, rutin and the absence of (−)-epigallocatechin (Figure 4d). Moreover, *Astragalus* and *Codonopsis* honeys were distinguished from other honeys by a higher content of calycosin and formononetin, and *Astragalus* honeys showed high correlations with a higher content of isorhamnetin and no detection of (−)-epigallocatechin, whereas *Codonopsis* honey had a higher content of myricetin, rutin, gallic acid and TPC, which was used for discriminating (Figure 4e,f).

## 3. Materials and Methods

### 3.1. Honey Samples

A total of 66 honey samples from 17 acacia (*Robinia pseudoacacia* L.), 17 *Vitex* (*Vitex negundo* var. *heterophylla* Rehd.), 17 linden (*Tiliaamurensis* Rupr.), four rapeseed (*Brassica campestris* L.), five *Astragalus* (*Astragalus membranaceus* (Fisch.) Bunge) and six *Codonoposis* (*Codonopsis pilosula* (Franch.) Nannf.) were collected from Beijing, Shandong, Hebei, Jiangxi, Hubei, Jiangsu, Sichuan, Zhejiang, Heilongjiang, Jilin and Changbai Mountains in China, from April 2015 to April 2017. More than 500 g of each honey sample was collected from beekeepers and different producers, and stored at 4 °C.

### 3.2. Chemicals

Analytical standards of 3,4-dihydroxybenzoic acid (98.2%), chlorogenic acid (96%), 4-hydroxy-benzoic acid (99.7%), caffeic acid (99.2%), syringic acid (96.4%), 3-hydroxybenzoic acid (99.8%), *p*-coumaric acid (99.6%), genistin (98.3%), sinapic acid (98%), ferulic acid (99.6%), quercetrin (98.34%), 2-hydroxycinnamic acid (99.8%), fisetin (98.2%), myricetin (99.5%), ononin (100.0%), salicylic acid (99.9%), morin hydrate (90%), (±)-abscisic acid (99.3%), luteolin (98.34%), calycosin (100.0%), quercetin (96%), apigenin (99.6%), naringenin (96.2%), genistein (99.0%), kaempferol (99.1%), hesperetin (99%), chrysin (98.64%), pinocembrin (99%) were purchased from the Sigma-Aldrich Co. (St. Louis, MO, USA); (−)-epigallocatechin (98.25%), vitexin (100.0%), hesperidin (98%), isorhamnetin (98.12%), isoferulic acid (98.10%), formononein (98.02%) were purchased from Aladdin-E. (Shanghai, China); gallic acid (89.9%), rutin (91.9%), baicalin (93.9%) were purchased from the National Institute for Food and Drug Control (Beijing, China); calycosin-7-*O*-β-d-glucoside (99.0%) was purchased from Yongjian Pharmaceutical Co. Ltd. (Taizhou, Jiangsu, China). Stock standard solutions of each compound were prepared by dissolving the analytical standard in methanol to a concentration at 1 mg mL^−1^, with the exception of isorhamnetin, apigenin, hesperedin and genistin which were dissolved by dimethyl sulfoxide. All solutions were stored at 4 °C. An intermediate solution containing all standard compounds (1 μg mL^−1^) was prepared in methanol, which was diluted to different levels for calibration curves and validation experiments.

Folin-Ciocalteu reagent, sodium carbonate, 2,2-diphenyl-1-picrylhydrazyl (DPPH), L-ascorbic acid, 2,2′-azino-bis(3-etyllbenzothiazoline-6-sulfonic acid) diammonium salt (ABTS), potassium persulfate and phosphate buffer solution (1.0 mol L^−1^, pH 7.4 at 25 °C) and dimethyl sulfoxide were purchased from the Sigma Aldrich Co. LC/MS-grade formic acid, HPLC-grade methanol and acetonitrile were obtained from the Fisher Scientific Inc. (Geel, Belgium). Hydrochloric acid (1.004 mol/L at 20 °C) was from the National Chemical Reagent Quality Inspection Center (Beijing, China). Ultra-pure water was produced by a Millipore water purification system. The Oasis HLB cartridge (6cc, 500 mg) was supplied by Waters Corporation (Milford, MA, USA).

### 3.3. Sample Preparation

Each honey sample (10 g) was mixed with acidified water (50 mL), which was adjusted to pH 2 with calibrated hydrochloric acid for the RP SPE cartridges. The fluid sample was centrifuged at 14,000× *g* for 5 min to remove the solid particles. The supernatant sample was loaded onto a methanol-conditioned cartridge. Then, the cartridge was rinsed with 50 mL of acidified ultrapure water (pH2) to remove saccharides and other polar compounds. Phenolic and flavonoid compounds absorbed on the cartridge were eluted with 30 mL of methanol. The methanol solution was evaporated to dryness by a rotary evaporator (IKA, Staufen, Germany) at 40 °C, and the residue was reconstituted in 1 mL of methanol. All solutions samples were filtered through a 0.20 μm syringe filter from Waters Corporation prior to UPLC injection.

### 3.4. UPLC-MS/MS Instrumentation

Chromatographic experiment was performed on a Waters ACQUITY UPLC system. Separation was achieved on a Waters ACQUITY UPLC HSS T3 column (2.1 × 100 mm, 1.8 μm) using a mobile phase that consisted of 0.1% formic acid in water (A) and acetonitrile (B) with the following gradient program (*v*/*v*): 0–1.00 min, A: 97%; 1.00–18.00 min, A: 97–10%; 18.00–20.00 min, A: 10%; 20.00–20.10 min, A: 10–0%; 20.10–23.00 min, A: 0%; 23.00–23.10 min, A: 0–97%; 23.10–28.00 min, A: 97%. The flow rate of the mobile phase was 0.3 mL/min, the column temperature was set at 35 °C, and the injection volume was 1 μL.

For the mass spectrometric analysis, a Waters Xevo TQ-S instrument equipped with an electrospray ionization source, operating in the positive or negative ionization modes, was set with the following parameters: capillary voltage: 3.00 kv; source temperature: 150 °C and desolvation temperature: 500 °C; the nitrogen gas flows were 650 L/h and 150 L/h for the desolvation and cone gases, respectively; argon was employed as the collision gas with a flow rate of 0.25 mL min^−1^. Table 1 shows the instrument settings optimized for product ions of each compound. Acquisition was performed in the multiple reaction monitoring (MRM) mode, and Masslynx 4.1 (Waters) was used for the data acquisition and processing.

### 3.5. Determination of Total Phenolic Content (TPC)

The Folin-Ciocalteu method was used to determine TPC in honey [25]. Thirty μL of honey solution (16 % *w*/*v* in water) was mixed with 0.2 N Folin-Ciocalteu reagent (150 μL) followed by the addition of 120 μL (75 g/L) of sodium carbonate in 96-well plates. The mixture was incubated at ambient temperature for 2 h and the absorbance of reaction mixture was measured at 760 nm against methanol blank by using a SpectraMax i3x Multi-Mode Detection Platform (Molecular Devices, Sunnyvale, CA,, USA). TPC was determined by comparing to the standard curve using gallic acid in the concentration range of 1.24–124.55 μg/mL. The results were expressed as mg of gallic acid equivalents (mg GAE)/100 g of honey.

### 3.6. DPPH Assay

A sample (1.6 g) was dissolved in water (1 mL) and diluted to 16 % *w*/*v* in distilled water. Thereafter, DPPH reagent solution (225 μL, 0.02 mg/mL in methanol) was added to honey solution (15 μL) in 96-well plates, then the mixtures were kept in the dark for 15 min at room temperature. The absorbance of each mixture was measured at 517 nm against methanol blank by using the SpectraMax i3x Multi-Mode Detection Platform. The radical scavenging activity (RSA) of DPPH expressed as inhibition% was calculated from the following equation [26]:

Inhibition% = [(Abs control − Abs sample)/Abs control] × 100%(2)

where Abs control is the absorbance of the mixture (225 μL of DPPH and 15 μL of methanol) at 517 nm and Abs sample is the absorbance of sample with DPPH at 517 nm. The antioxidant content in terms of antioxidant equivalent ascorbic acid content (AEAC) was determined and expressed as mg of ascorbic acid equivalent antioxidant content per 100 g of honey (mg AEAC/100 g) using standard curve of ascorbic acid (5.19–51.87 μg/mL).

### 3.7. ABTS Assay

ABTS + radical scavenging assay was performed as follows [27]. The cation radical ABTS+ was obtained in the reaction of 7 mmol L^−1^ stock solution of 2,2′-azino-bis(3-etyllbenzothiazoline-6-sulfonic acid) diammonium salt (ABTS) with 2.45 mmol L^−1^ potassium persulfate solution. The mixture was left to stand for 16 h in the dark at ambient temperature. Prior to analysis, the ABTS+ solution was diluted with phosphate buffer (1.0 M, pH 7.4) to produce a solution with an absorbance of 0.700 ± 0.010 at 734 nm. 1.6 g of sample was dissolved in 1 mL of water and diluted to 16% *w*/*v* in distilled water, then 50 μL of honey solution was mixed with 200 μL of ABTS + cation radical solution and after 6 min absorbance was measured at 734 nm by using the SpectraMax i3x Multi-Mode Detection Platform. The radical scavenging activity (RSA) of ABTS + expressed as inhibition% was calculated from the following equation:

Inhibition% = [(Abs control − Abs sample)/Abs control] × 100%.(3)

where Abs control is the absorbance of control (200 μL ABTS + and 50 μL water) at 734 nm and Abs sample is the absorbance of sample with ABTS+ at 734 nm. The antioxidant content in terms of antioxidant equivalent ascorbic acid content (AEAC) was determined by the curve of concentration of AEAC via inhibition of AEAC. The antioxidant content was expressed as mg of ascorbic acid equivalent antioxidant content per 100 g of honey (mg AEAC/100 g) using standard curve of ascorbic acid (5.19–51.87 μg/mL).

### 3.8. Statistical Analysis

Analyses were determined in duplicate and the data are presented as mean ± standard deviation. Pearson’s correlation (*p* < 0.01) observed between total phenolic content (TPC) and antioxidant activity was carried out by SPSS 16.0 (SPSS, Inc., Chicago, IL, USA). The obtained data set (X matrix) contained the contents of phenolic, flavonoids and abscisic acid measured by UPLC-MS/MS, as well as TPC and antioxidant properties. The data matrix was transferred into SIMCA-P+ software (v 13.0, Umetrics, Umeå, Sweden), where principal component analysis (PCA), hierarchical cluster analysis (HCA), partial least squares-discriminant analysis (PLS-DA) and orthogonal partial least squares-discriminant analysis (OPLS-DA) were conducted. Prior to multivariate analysis, the data were verified by Hotelling’s T2 range algorithm in 95% confidence interval, log-transformed and scaled using Pareto and unit variance (UV) scaling. The supervised OPLS-DA models were validated by means of cross-validation analysis of a permutation test with 200 cycles by PLS-DA, where R2 and Q2 were calculated as the goodness of fit and the predictive capability of the model, respectively [28].

## 4. Conclusions

A reproducible and sensitive UPLC-MS/MS method was optimized and validated for simultaneous determination of 38 phenolics, flavonoids and (±)-abscisic acid in honeys. The solid phase extraction method used provided excellent recoveries. For the first time, formononetin, calycosin, ononin and calycosin-7-*O*-β-d-glucoside were identified and quantified in honey, among which formononetin and calycosin can be used for characteristic markers for *Astragalus* and *Codonopsis* honeys. Variable amounts of phenolic and flavonoids in honeys from different floral origins indicated correlations with discriminative antioxidant competences and potential floral markers. The distribution of various phenolics and flavonoids, and antioxidant activities discriminated acacia, *Vitex*, linden, rapeseed, *Astragalus* and *Codonopsis* honeys using PCA, HCA and OPLS-DA analysis, and the prediction evaluation using PLS-DA analysis indicated that the phenolic and flavonoid profiles as well as antioxidant competence could be used for honey identification as an alternative approach. However, the collection of monofloral honeys from traditional Chinese medicines is difficult, leading to difficult discrimination, therefore exploration of compounds referred to secondary metabolites from nectar plants are recommended.

## Figures and Tables

**Table 1:** Mass spectrometric conditions for each compound.

Code	Compound	*t*_R_ (min)	Ion Mode	MRM (*m*/*z*)	Cone (eV)	Collision (eV)	MS Segment Time (min)
**1**	Gallic acid	3.22	ES−	169 → 125	42	14	0.0–5.0
**2**	3,4-Dihydroxybenzoic acid	4.31	ES−	153 → 109	54	14	0.0–5.0
**3**	(−)-Epigallocatechin	4.94	ES+	306 → 139	12	16	0.0–5.0
**4**	Chlorogenic acid	4.99	ES−	353 → 191	40	26	4.0–6.0
**5**	4-Hydroxybenzoic acid	5.26	ES−	137 → 93	30	15	4.5–6.5
**6**	Caffeic acid	5.63	ES−	179 → 135	42	18	5.0–6.0
**7**	Syringic acid	5.68	ES+	199 → 140	2	16	5.0–6.0
**8**	3-Hydroxybenzoic acid	5.86	ES−	137 → 93	30	15	4.5–6.5
**9**	Rutin	6.28	ES−	609 → 300	90	30	6.0–7.0
**10**	Calycosin-7-*O*-β-d-glucoside	6.32	ES+	447 → 285	45	15	6.0–7.0
**11**	Vitexin	6.36	ES+	433 → 415	30	18	6.0–7.0
**12**	*p*-Coumaric acid	6.58	ES−	163 → 119	46	18	6.0–8.0
**13**	Genistin	6.77	ES+	433 → 271	40	10	6.0–7.0
**14**	Sinapic acid	6.78	ES+	225 → 175	50	16	6.0–7.0
**15**	Ferulic acid	6.85	ES+	195 → 177	26	10	6.0–7.5
**16**	Isoferulic acid	7.03	ES+	195 → 177	26	10	6.0–7.5
**17**	Hesperedin	7.06	ES−	609 → 301	54	26	6.5–7.5
**18**	Quercetrin	7.08	ES−	447 → 300	30	25	6.5–8.0
**19**	2-Hydroxycinnamic acid	7.69	ES−	163 → 119	46	18	6.0–8.0
**20**	Fisetin	7.72	ES+	287 → 137	56	35	7.0–8.0
**21**	Myricetin	7.74	ES+	319 → 153	16	24	7.0–8.0
**22**	Baicalin	7.76	ES+	447 → 271	45	12	7.0–8.0
**23**	Ononin	7.78	ES+	431 → 269	40	15	7.0–8.0
**24**	Salicylic acid	8.23	ES-	137 → 93	46	14	7.0–9.0
**25**	Morin	8.29	ES+	303 → 153	6	35	7.8–9.0
**26**	(±)-Abscisic acid	8.43	ES−	263 → 153	2	10	8.0–9.0
**27**	Luteolin	8.76	ES+	287 → 153	32	32	8.0–9.5
**28**	Calycosin	8.77	ES+	285 → 270	30	25	8.0–9.5
**29**	Quercetin	8.85	ES+	303 → 153	72	28	8.0–9.5
**30**	Apigenin	9.70	ES+	271 → 91	76	38	9.0–10.0
**31**	Naringenin	9.75	ES+	273 → 153	48	22	9.0–10.0
**32**	Genistein	9.79	ES+	271 → 91	76	38	9.0–10.0
**33**	Kaempferol	9.92	ES+	287 → 153	56	30	9.0–10.5
**34**	Hesperetin	10.02	ES+	303 → 153	64	24	9.0–11.0
**35**	Isorhamnetin	10.03	ES−	315 → 300	64	22	9.0–11.0
**36**	Formononein	10.75	ES+	269 → 213	45	25	10.0–11.0
**37**	Chrysin	11.87	ES+	255 → 153	40	28	11.0–15.0
**38**	Pinocembrin	12.10	ES+	257 → 153	42	22	11.0–15.0

**Table 2:** Method validation results for each compound.

Code	Compounds	Linear Range (μg L^−1^)	Regression Equation	R	LOD (μg L^−1^)	LOQ (μg L^−1^)	Recovery (%) (*n* = 3)	RSD (%) (*n* = 6)	
								Intra-Day	Inter-Day
**1**	Gallic acid	6.29–1259	*Y* = 384.9*x* − 1408.5	0.9996	1.01	6.29	85.86	5.64	5.08
**2**	3,4-Dihydroxybenzoic acid	11.22–1122	*Y* = 244.7*x* − 241.7	0.9997	7.01	11.22	56.07	4.72	3.67
**3**	(−)-Epigallocatechin	19.88–994	*Y* = 27.5*x* − 29.8	0.9987	6.21	19.88	72.17	8.21	4.18
**4**	Chlorogenic acid	1.21–1217	*Y* = 331.8*x* − 1137.0	0.9996	0.12	0.48	84.87	7.60	6.54
**5**	4-Hydroxybenzoic acid	30.65–982	*Y* = 126.9*x* + 236.5	0.9998	6.13	30.65	103.58	4.30	4.44
**6**	Caffeic acid	9.11–1458	*Y* = 912.4*x*-344.0	0.9986	4.56	9.11	78.88	4.44	4.91
**7**	Syringic acid	5.12–1024	*Y* = 672.3*x* + 881.8	0.9992	3.20	5.12	90.90	3.20	6.85
**8**	3-Hydroxybenzoic acid	113.97–1139	*Y* = 44.9*x* + 49.1	0.9980	28.49	113.97	89.87	4.51	6.48
**9**	Rutin	5.40–1081	*Y* = 82.3*x* − 270.3	0.9999	1.08	5.40	94.51	7.36	1.50
**10**	Calycosin-7-*O*-β-d-glucoside	1.02–1019	*Y* = 5920.2*x* + 5595.9	0.9988	0.01	0.03	89.85	7.28	6.98
**11**	Vitexin	2.55–1018	*Y* = 1702.1*x* + 1415.0	0.9989	0.51	2.55	96.86	7.07	7.71
**12**	*p*-Coumaric acid	3.81–1524	*Y* = 555.8*x* + 450.2	0.9995	1.27	3.81	81.75	2.58	5.87
**13**	Genistin	1.01–1016	*Y* = 2230.6*x* + 2080.8	0.9995	0.02	0.05	85.84	5.88	3.85
**14**	Sinapic acid	34.22–1369	*Y* = 171.2*x* + 252.5	0.9988	11.41	34.22	88.39	6.86	5.78
**15**	Ferulic acid	26.67–1067	*Y* = 1416.4*x* + 1173.9	0.9991	8.89	26.67	81.66	2.70	5.27
**16**	Isoferulic acid	28.41–1153	*Y* = 2096.76*x* + 17,848.8	0.9993	7.10	28.41	90.48	2.77	3.36
**17**	Hesperedin	4.07–1304	*Y* = 227.8*x* + 1535.5	0.9994	0.22	0.65	93.31	7.58	2.62
**18**	Quercetrin	7.65–1020	*Y* = 153.2*x* − 301.1	0.9998	5.10	7.65	90.14	6.30	6.36
**19**	2-Hydroxycinnamic acid	14.54–1454	*Y* = 151.8*x* + 244.5	0.9997	4.54	14.54	97.04	2.97	3.46
**20**	Fisetin	0.24–1184	*Y* = 3236.1*x* − 7687.7	0.9990	0.12	0.24	106.76	8.05	5.92
**21**	Myricetin	1.02–1019	*Y* = 877.6*x* − 4330.5	0.9968	0.41	1.02	74.15	8.55	5.27
**22**	Baicalin	1.12–1195	*Y* = 2314.3*x* − 3403.1	0.9985	0.02	0.06	100.78	5.76	3.21
**23**	Ononin	0.89–892	*Y* = 6642.5*x* + 12,287.6	0.9979	0.01	0.03	89.64	5.17	4.34
**24**	Salicylic acid	11.23–1123	*Y* = 413.6*x* + 1044.2	0.9991	0.90	11.23	99.96	1.43	8.68
**25**	Morin	0.66–1108	*Y* = 1371.3*x* − 91.9	0.9998	0.22	0.66	80.95	3.61	4.32
**26**	(±)-Abscisic acid	1.17–1166	*Y* = 192.9*x* + 117.0	0.9995	0.12	1.17	104.04	3.56	6.74
**27**	Luteolin	0.28–1387	*Y* = 6169.6*x* − 7098.0	0.9972	0.14	0.28	102.43	3.73	3.47
**28**	Calycosin	0.03–917	*Y* = 5110.9*x* + 10,922	0.9978	0.01	0.03	93.58	4.68	5.22
**29**	Quercetin	1.04–1042	*Y* = 1260.5*x* − 3603.3	0.9988	0.10	0.62	78.42	6.34	4.58
**30**	Apigenin	0.39–970	*Y* = 2276.1*x* + 2335.7	0.9988	0.09	0.39	91.11	7.66	8.05
**31**	Naringenin	0.12–1201	*Y* = 8620.0*x* + 15367	0.9970	0.04	0.12	83.08	4.32	3.56
**32**	Genistein	0.10–1018	*Y* = 2902.9*x* + 3037.9	0.9992	0.04	0.10	105.20	9.31	5.77
**33**	Kaempferol	3.18–1017	*Y* = 2343.7*x* − 1116.31	0.9992	0.05	0.40	99.73	6.81	6.57
**34**	Hesperetin	0.13–1319	*Y* = 7416.7*x* + 10,744.2	0.9988	0.04	0.13	93.31	4.20	2.87
**35**	Isorhamnetin	0.39–988	*Y* = 1309.5*x* − 346.1	0.9995	0.10	0.39	67.55	7.23	5.42
**36**	Formononein	0.03–1166	*Y* = 2685.3*x* + 6750.1	0.9977	0.01	0.03	106.66	5.30	5.56
**37**	Chrysin	0.11–1081	*Y* = 6481.2*x* + 11,968.2	0.9992	0.03	0.11	92.25	2.87	4.66
**38**	Pinocembrin	0.04–1028	*Y* = 8341.6*x* + 13,889.1	0.9981	0.01	0.04	75.5	1.87	5.17

**Table 3:** Content of phenolic and flavonoid compounds in acacia, *Vitex* and linden honey samples (ng g^−1^).

Compound	Acacia (*n* = 17)			*Vitex* (*n* = 17)			Linden (*n* = 17)		
	Mean ± SD	Min	Max	Mean ± SD	Min	Max	Mean ± SD	Min	Max
Ferulic acid	41.99 ± 13.82	12.08	70.26	69.75 ± 36.65	23.82	155.76	79.87 ± 45.81	9.47	169.16
Isoferulic	35.28 ± 22.58	9.46	86.54	46.93 ± 29.98	15.19	121.31	89.85 ± 57.17	13.98	203.17
Syringic acid	10.10 ± 6.54	3.38	28.05	15.47 ± 14.05	2.91	48.33	10.87 ± 7.74	3.82	38.62
Sinapic acid	4.74 ± 3.57	1.13	15.30	3.25 ± 3.64	ND	15.33	ND	ND	ND
Chrysin	44.64 ± 41.51	5.56	139.81	95.45 ± 83.36	13.30	320.00	50.28 ± 21.41	13.82	91.08
Pinocembrin	26.64 ± 23.11	1.98	98.67	99.36 ± 53.36	20.59	175.25	92.89 ± 46.30	20.34	198.92
Formononetin	2.20 ± 1.96	ND	6.55	0.17 ± 0.14	ND	0.39	0.18 ± 0.42	ND	1.49
Apigenin	21.25 ± 17.50	3.96	66.66	175.63 ± 89.84	56.67	358.91	26.97 ± 36.34	2.55	154.79
Genistein	1.30 ± 1.49	0.03	5.15	3.33 ± 2.58	ND	6.92	0.31 ± 0.49	ND	1.43
Naringenin	24.66 ± 15.97	7.44	54.27	20.23 ± 12.14	2.59	48.48	9.94 ± 4.91	2.49	18.78
Calycosin	1.06 ± 0.62	ND	2.53	ND	ND	ND	ND	ND	ND
Luteolin	42.57 ± 53.90	3.25	233.03	146.22 ± 56.75	56.70	268.95	16.4 0 ± 10.65	2.95	45.97
Kaempferol	174.48 ± 132.77	10.51	556.02	189.86 ± 119.25	10.27	387.24	71.27 ± 51.11	16.36	195.21
Hesperetin	4.26 ± 5.59	ND	14.25	6.10 ± 6.87	ND	23.59	13.41 ± 9.89	ND	32.54
Quercetin	77.41 ± 76.17	ND	249.71	68.81 ± 64.85	8.08	197.91	48.99 ± 47.07	11.95	157.27
(−)-Epigallocatechin	65.29 ± 33.45	5.46	122.15	18.04 ± 13.21	ND	60.20	78.56 ± 67.74	ND	268.22
Myricetin	ND	ND	ND	ND	ND	ND	4.04 ± 2.37	ND	8.97
Ononin	0.21 ± 0.25	ND	0.98	0.24 ± 0.18	ND	0.61	0.06 ± 0.09	ND	0.33
Genistin	0.25 ± 0.10	ND	0.46	0.17 ± 0.11	ND	0.36	ND	ND	ND
Vitexin	1.96 ± 1.41	ND	5.93	46.97 ± 15.86	19.81	87.29	3.38 ± 3.90	ND	14.66
Calycosin-7-*O*-β-d-glucoside	0.05 ± 0.03	ND	0.08	ND	ND	ND	ND	ND	ND
Baicalin	0.48 ± 0.10	0.37	0.74	0.44 ± 0.13	ND	0.64	1.67 ± 0.11	1.46	1.89
4-Hydroxybenzoic acid	402.21 ± 128.44	162.31	696.02	2543.25 ± 794.81	1229.77	4418.46	576.37 ± 178.41	356.80	1079.21
3-Hydroxybenzoic acid	<LOQ	<LOQ	<LOQ	10.74 ± 5.65	<LOQ	18.76	<LOQ	<LOQ	<LOQ
Gallic acid	10.70 ± 7.55	2.76	29.23	35.05 ± 30.29	6.52	137.90	6.42 ± 10.01	ND	37.93
*p*-Coumaric acid	31.43 ± 11.37	8.77	47.01	119.20 ± 48.81	38.44	216.92	67.61 ± 27.92	3.67	127.34
Salicylic acid	54.87 ± 18.46	25.78	92.00	50.16 ± 16.88	23.89	91.88	58.38 ± 26.62	31.59	144.83
Caffeic acid	58.37 ± 25.43	19.62	119.87	772.60 ± 366.94	134.31	1758.10	338.40 ± 272.01	29.06	1116.66
(±)-Abscisic acid	510.86 ± 158.42	212.75	741.68	192.60 ± 165.20	65.09	746.47	357.77 ± 171.94	9.54	770.10
Isorhamnetin	29.94 ± 29.47	ND	101.83	22.13 ± 13.14	ND	46.20	2.55 ± 1.55	ND	6.80
3,4-Dihydroxybenzoic acid	49.49 ± 15.05	24.01	76.83	233.63 ± 60.63	137.06	369.53	822.22 ± 516.47	177.86	2424.19
Chlorogenic acid	80.84 ± 34.25	37.62	163.24	3171.45 ± 1462.30	1398.61	6635.87	13.89 ± 18.46	ND	58.59
Rutin	4.46 ± 2.47	1.44	9.24	7.09 ± 8.22	1.07	33.38	3.10 ± 1.81	1.13	7.86
Quercetrin	0.65 ± 0.41	0.30	1.66	4.99 ± 12.63	0.38	52.03	1.85 ± 2.90	0.42	12.85
Hesperedin	17.22 ± 24.08	0.03	75.95	2.19 ± 1.75	ND	5.89	0.74 ± 0.57	ND	1.89
Fisetin	ND	ND	ND	ND	ND	ND	ND	ND	ND
Morin	ND	ND	ND	ND	ND	ND	ND	ND	ND
2-Hydroxybenzoic acid	ND	ND	ND	ND	ND	ND	ND	ND	ND

**Table 4:** Content of phenolic and flavonoid compounds in rapeseed, *Astragalus* and *Codonopsis* honey samples (ng g^−1^).

Compound	Rapeseed (*n* = 4)			*Astragalus* (*n* = 5)			*Codonopsis* (*n* = 6)		
	Mean ± SD	Min	Max	Mean ± SD	Min	Max	Mean ± SD	Min	Max
Ferulic acid	23.99 ± 15.44	14.59	47.05	27.53 ± 10.25	17.52	42.68	38.71 ± 21.13	20.63	65.74
Isoferulic	20.29 ± 12.35	12.33	38.53	27.37 ± 15.01	5.94	41.83	25.76 ± 12.22	4.79	39.72
Syringic acid	73.29 ± 45.62	44.48	140.82	11.31 ± 5.30	5.04	19.36	23.87 ± 10.45	14.08	43.51
Sinapic acid	4.92 ± 2.67	2.41	7.94	1.97 ± 0.79	0.69	2.85	2.61 ± 1.66	1.02	5.33
Chrysin	65.05 ± 28.72	43.51	107.39	80.72 ± 29.51	62.97	133.11	118.73 ± 63.67	73.06	245.76
Pinocembrin	33.88 ± 37.16	6.61	86.46	63.39 ± 21.02	41.10	94.04	79.61 ± 28.16	45.89	128.01
Formononetin	ND	ND	ND	12.66 ± 6.80	5.35	19.52	8.86 ± 5.83	3.15	19.52
Apigenin	25.63 ± 30.08	6.54	70.47	226.60 ± 372.61	28.70	887.47	260.07 ± 86.76	174.00	403.28
Genistein	ND	ND	ND	3.79 ± 2.30	1.24	6.27	2.29 ± 2.86	ND	7.46
Naringenin	21.67 ± 9.00	11.64	31.58	30.54 ± 17.97	12.92	55.47	16.44 ± 2.42	13.51	20.21
Calycosin	ND	ND	ND	23.80 ± 18.40	6.58	45.95	9.17 ± 8.11	0.90	20.11
Luteolin	3.79 ± 0.97	3.00	5.09	61.17 ± 17.50	43.47	87.23	46.21 ± 29.59	20.58	104.08
Kaempferol	665.28 ± 192.31	504.93	941.61	675.47 ± 525.43	263.00	1570.31	637.91 ± 645.06	129.15	1867.88
Hesperetin	0.28 ± 0.40	0.02	0.87	8.50 ± 8.03	1.85	19.32	6.00 ± 4.33	3.55	14.71
Quercetin	362.00 ± 370.27	ND	874.94	441.28 ± 416.47	15.91	1054.61	347.14 ± 178.68	66.39	534.60
(−)-Epigallocatechin	ND	ND	ND	ND	ND	ND	11.77 ± 12.96	ND	25.69
Myricetin	4.91 ± 1.17	3.21	5.80	7.52 ± 3.82	3.70	12.84	14.06 ± 5.20	8.06	20.95
Ononin	0.14 ± 0.20	ND	0.44	0.35 ± 0.05	0.29	0.43	0.54 ± 0.14	0.43	0.77
Genistin	ND	ND	ND	ND	ND	ND	ND	ND	ND
Vitexin	2.20 ± 3.55	ND	7.44	3.15 ± 2.01	1.31	6.36	4.64 ± 7.04	ND	18.67
Calycosin-7-*O*-β-d-glucoside	ND	ND	ND	0.31 ± 0.69	ND	1.54	0.04 ± 0.09	ND	0.21
Baicalin	0.92 ± 0.02	0.90	0.94	0.93 ± 0.03	0.90	0.97	0.85 ± 0.06	0.77	0.94
4-Hydroxybenzoic acid	1211.08 ± 703.69	774.67	2255.48	863.44 ± 156.21	620.75	988.20	924.22 ± 172.17	652.33	1159.95
3-Hydroxybenzoic acid	<LOQ	<LOQ	<LOQ	<LOQ	<LOQ	<LOQ	<LOQ	<LOQ	<LOQ
Gallic acid	14.23 ± 18.22	2.41	41.25	40.62 ± 34.52	18.89	100.55	111.84 ± 61.10	45.21	214.12
*p*-Coumaric acid	107.74 ± 70.29	57.39	206.72	66.90 ± 28.57	33.33	112.58	111.50 ± 49.50	55.37	199.70
Salicylic acid	91.91 ± 58.38	52.12	177.00	86.24 ± 23.46	53.31	118.07	95.07 ± 23.93	52.14	116.31
Caffeic acid	23.63 ± 22.47	6.52	56.54	107.88 ± 30.05	60.78	142.53	166.13 ± 125.49	56.49	407.22
(±)-Abscisic acid	160.04 ± 126.25	88.00	348.49	253.81 ± 164.57	90.34	487.75	426.48 ± 182.31	246.66	690.30
Isorhamnetin	54.89 ± 30.66	28.88	92.97	124.46 ± 87.62	20.15	229.85	90.99 ± 29.99	35.63	115.84
3,4-Dihydroxybenzoic acid	150.52 ± 66.51	61.37	218.47	126.77 ± 46.68	60.90	185.77	370.52 ± 195.02	155.20	686.77
Chlorogenic acid	3.85 ± 2.57	ND	5.19	67.38 ± 77.60	ND	156.86	76.56 ± 90.42	ND	224.27
Rutin	0.94 ± 1.11	ND	2.54	3.97 ± 3.18	ND	7.64	22.62 ± 17.19	8.54	53.58
Quercetrin	0.99 ± 1.37	0.25	3.04	2.17 ± 1.20	0.63	3.40	5.33 ± 3.65	1.44	11.56
Hesperedin	0.95 ± 1.10	ND	1.91	6.25 ± 6.87	ND	17.11	4.72 ± 8.82	ND	22.15
Fisetin	ND	ND	ND	ND	ND	ND	ND	ND	ND
Morin	ND	ND	ND	ND	ND	ND	ND	ND	ND
2-Hydroxybenzoic acid	ND	ND	ND	ND	ND	ND	ND	ND	ND

**Table 5:** Antioxidant properties of different honey varieties.

Parameter	Acacia (*n* = 17)	*Vitex* (*n* = 17)	Linden (*n* = 17)	Rapeseed (*n* = 4)	*Astragalus* (*n* = 5)	*Codonopsis* (*n* = 6)
TPC (mg GAE/100g)	11.04 ± 1.33	16.01 ± 2.76	17.26 ± 3.00	14.62 ± 1.29	15.77 ± 1.84	24.31 ± 2.32
DPPH-RSA (%)	10.56 ± 2.18	26.40 ± 9.73	32.76 ± 10.27	11.40 ± 4.18	22.19 ± 7.23	34.95 ± 6.98
DPPH-AEAC (mg AA/100 g)	4.61 ± 1.02	12.03 ± 4.41	14.85 ± 4.80	5.06 ± 2.11	8.97 ± 3.42	16.43 ± 2.95
ABTS-RSA (%)	66.10 ± 2.47	75.34 ± 4.60	80.62 ± 4.47	68.01 ± 6.08	70.84 ± 4.12	80.82 ± 5.22
ABTS-AEAC (mg AA/100 g)	29.52 ± 1.47	33.13 ± 2.04	34.70 ± 2.29	30.39 ± 1.69	31.28 ± 1.40	36.13 ± 1.22

Results are expressed as mean values ± standard deviations. GAE: Gallic Acid Equivalents; AEAC: Antioxidant Equivalent Ascorbic acid Content; DPPH RSA: 2,2-diphenyl-1-picrylhydrazyl Radical Scavenging Activity; ABTS RSA: 2,2′-azino-bis(3-etyllbenzothiazoline-6-sulfonic acid) diammonium salt Radical Scavenging Activity.

**Table 6:** Classification list for prediction based on the PLS-DA models performed by considering the training set samples.

Botanical Origin	Probability of Fitting the Models of Class Membership					
	Acacia	*Vitex*	Linden	Rape	*Astragalus*	*Codonopsis*
acacia	**1.04**	−0.06	−0.01	−0.04	−0.06	0.15
acacia	**1.02**	0.02	−0.05	0.04	−0.19	0.15
acacia	**0.70**	0.08	0.29	−0.13	0.22	−0.16
*Vitex*	−0.08	**0.98**	0.12	0.02	−0.02	0.02
*Vitex*	0.12	**0.75**	0.17	−0.06	−0.20	0.22
linden	0.02	−0.02	**1.07**	−0.02	0.04	−0.08
linden	−0.18	0.09	**0.95**	0.06	0.19	0.11
linden	−0.13	0.08	**0.85**	−0.00	0.21	−0.01
rape	0.19	0.02	0.16	0.42	0.31	−0.11
*Astragalus*	−0.01	0.05	−0.10	−0.05	**0.98**	0.14
*Codonopsis*	−0.01	0.18	−0.17	0.03	0.43	**0.62**

## Supplementary Materials

The following are available online at http://www.mdpi.com/1420-3049/23/5/1110/s1, Figure S1: Chromatograms of analyzed compounds, Figure S2: OPLS-DA score plot between acacia versus the rest, Figure S3: OPLS-DA score plot between vitex versus the rest, Figure S4: OPLS-DA score plot between linden versus the rest, Figure S5: OPLS-DA score plot between rapeseed versus the rest, Figure S6: OPLS-DA score plot between astragalus versus the rest, Figure S7: OPLS-DA score plot between codonopsis versus the rest, Table S1: Content of phenolic and flavonoid compounds in acacia honey samples (ng g^−1^), Table S2: Content of phenolic and flavonoid compounds in vitex honey samples (ng g^−1^), Table S3: Content of phenolic and flavonoid compounds in linden honey samples (ng g^−1^), Table S4: Content of phenolic and flavonoid compounds in rapeseed, astragalus and codonopsis honey samples (ng g^−1^).
