# Supplementary material for: Quantitative and Discriminative Evaluation of Contents of Phenolic and Flavonoid and Antioxidant Competence for Chinese Honeys from Different Botanical Origins
Source: Molecules. 2018 May 8;23(5):1110. doi: 10.3390/molecules23051110 (PMC6099688; doi:10.3390/molecules23051110)
Supplement: Supplementary file 1 [file molecules-23-01110-s001.pdf]

Supplementary Material

Quantitative and Discriminative Evaluation of Contents of Phenolic and Flavonoid and Antioxidant Competence for Chinese Honeys from Different Botanical Origins

Shi Shen *, Jingbo Wang, Qin Zhuo, Xi Chen, Tingting Liu and Shuang-Qing Zhang *

National Institute for Nutrition and Health, Chinese Center for Disease Control and Prevention, 29 Nanwei Rd, Beijing 100050, China; shenshi@ninh.chinacdc.cn (S.S.); wangjb@ninh.chinacdc.cn (J.W); zhuoqin@ninh.chinacdc.cn (Q.Z.); chenxi@ninh.chinacdc.cn (X.C.); Liutt@ninh.chinacdc.cn (T.L.)

* Correspondence: shenshi@ninh.chinacdc.cn (S.S.); zhangshq@hotmail.com (S.-Q.Z.); Tel.: +86-10-6623-7258 (S.S.); Tel.: +86-10-6623-7226 (S.-Q.Z.)

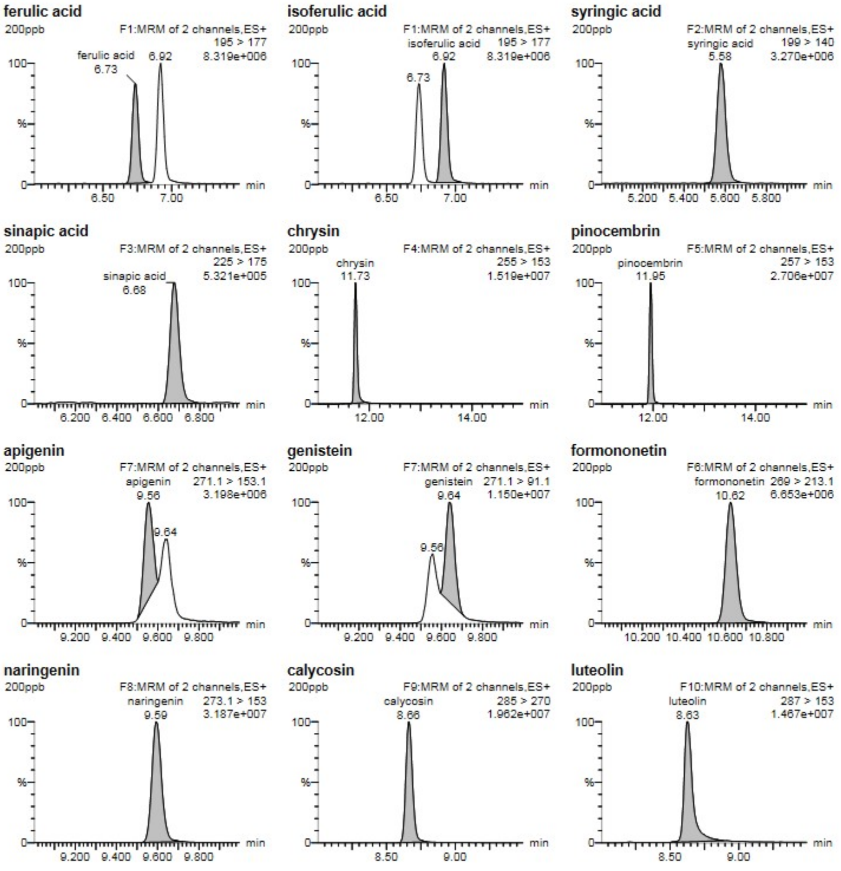

Figure S1. *Cont.*

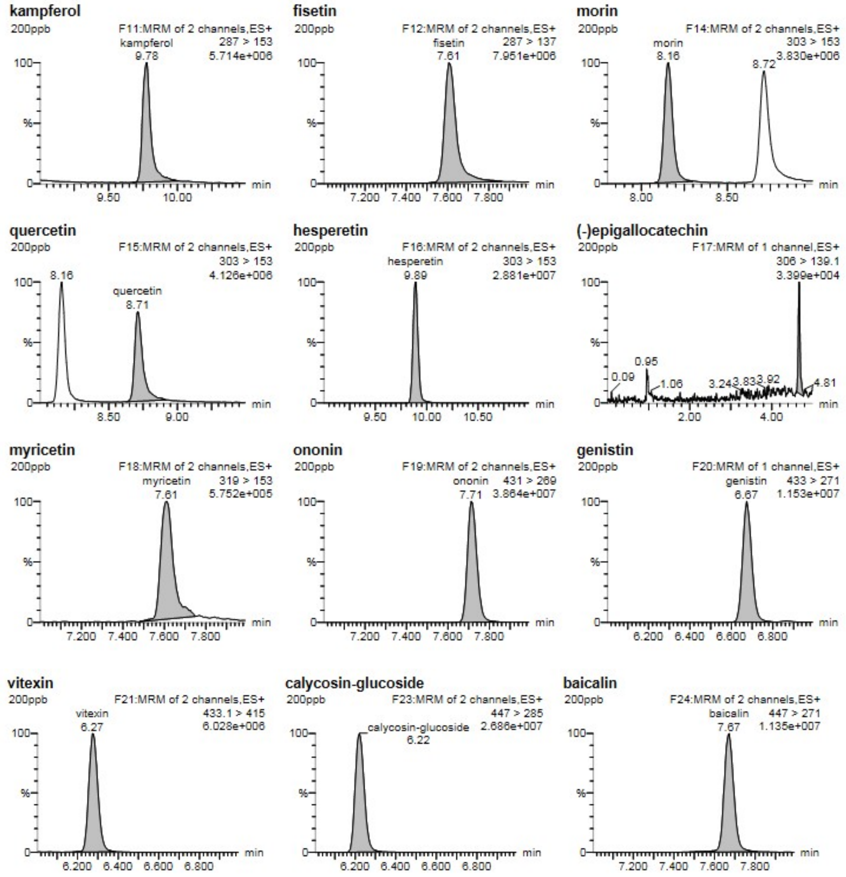

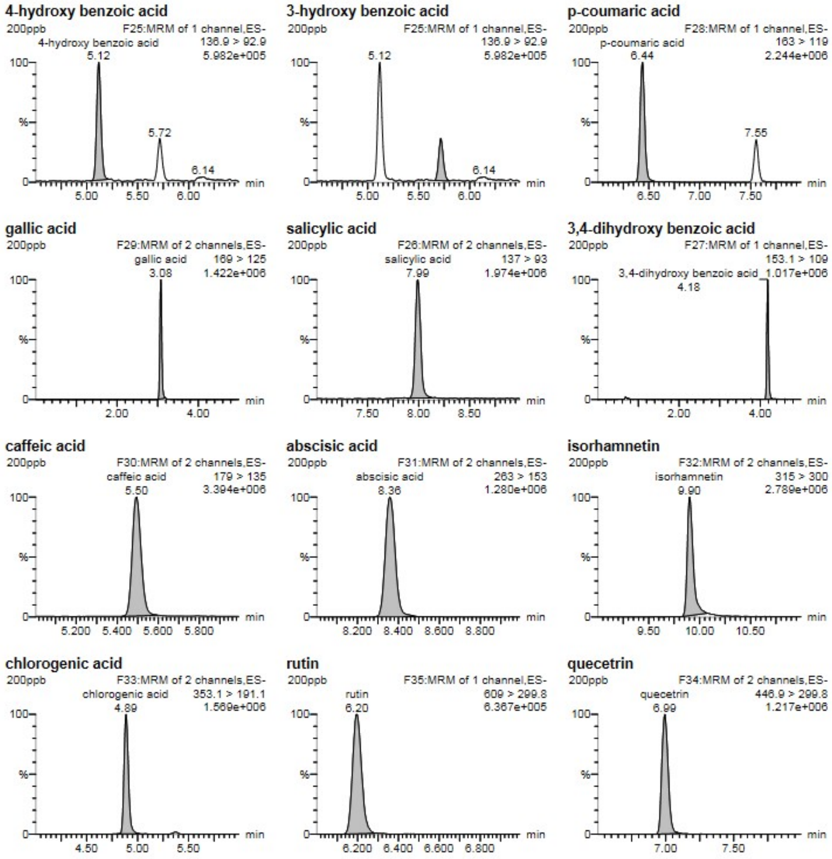

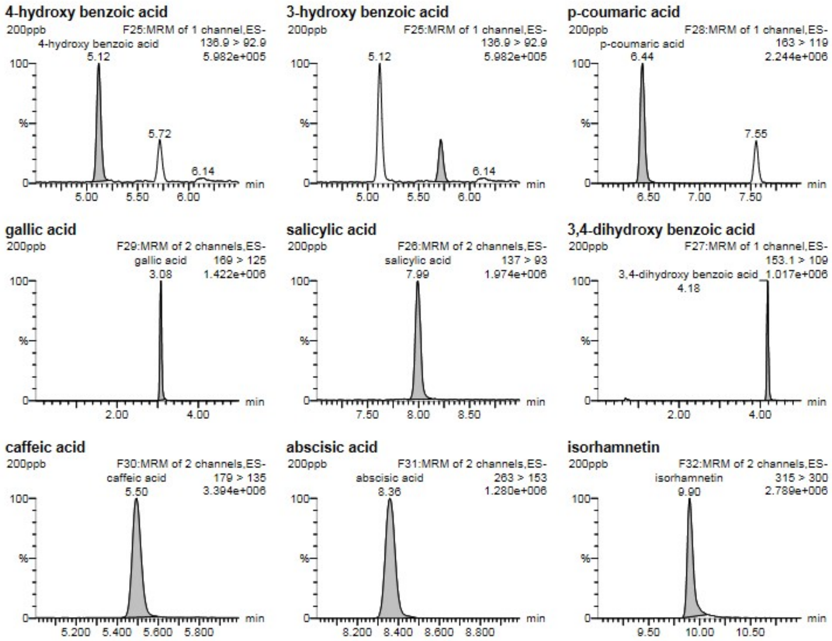
4-hydroxy benzoic acid
200ppb F25:MRM of 1 channel,ES-
4-hydroxy benzoic acid 136.9 > 92.9
5.982e+005
5.12
5.72
6.14
3-hydroxy benzoic acid
200ppb F25:MRM of 1 channel,ES-
136.9 > 92.9
5.982e+005
5.12
6.14
p-coumaric acid
200ppb F28:MRM of 1 channel,ES-
p-coumaric acid 163 > 119
2.244e+006
6.44
7.55
gallic acid
200ppb F29:MRM of 2 channels,ES-
gallic acid 169 > 125
3.08 1.422e+006
salicylic acid
200ppb F26:MRM of 2 channels,ES-
salicylic acid 137 > 93
7.99 1.974e+006
3,4-dihydroxy benzoic acid
200ppb F27:MRM of 1 channel,ES-
153.1 > 109
3,4-dihydroxy benzoic acid 1.017e+006
4.18
caffeic acid
200ppb F30:MRM of 2 channels,ES-
caffeic acid 179 > 135
5.50 3.394e+006
abscisic acid
200ppb F31:MRM of 2 channels,ES-
abscisic acid 263 > 153
8.36 1.280e+006
isorhamnetin
200ppb F32:MRM of 2 channels,ES-
isorhamnetin 315 > 300
9.90 2.789e+006

Figure S1. *Cont.*

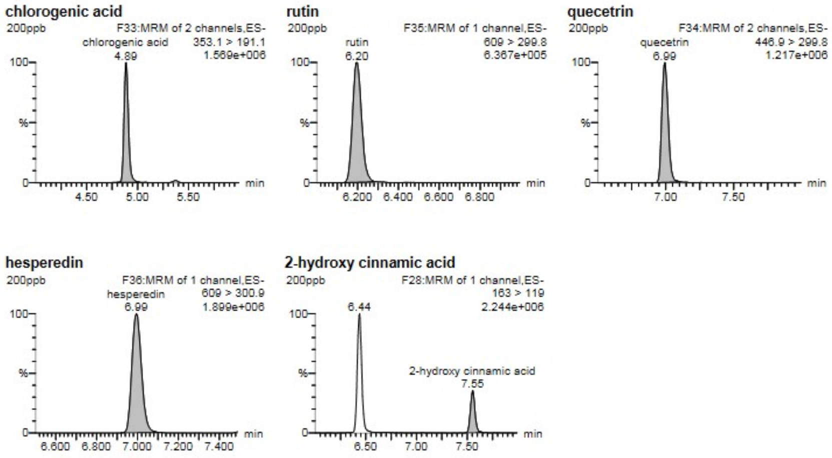

Figure S1. Chromatograms of analyzed compounds

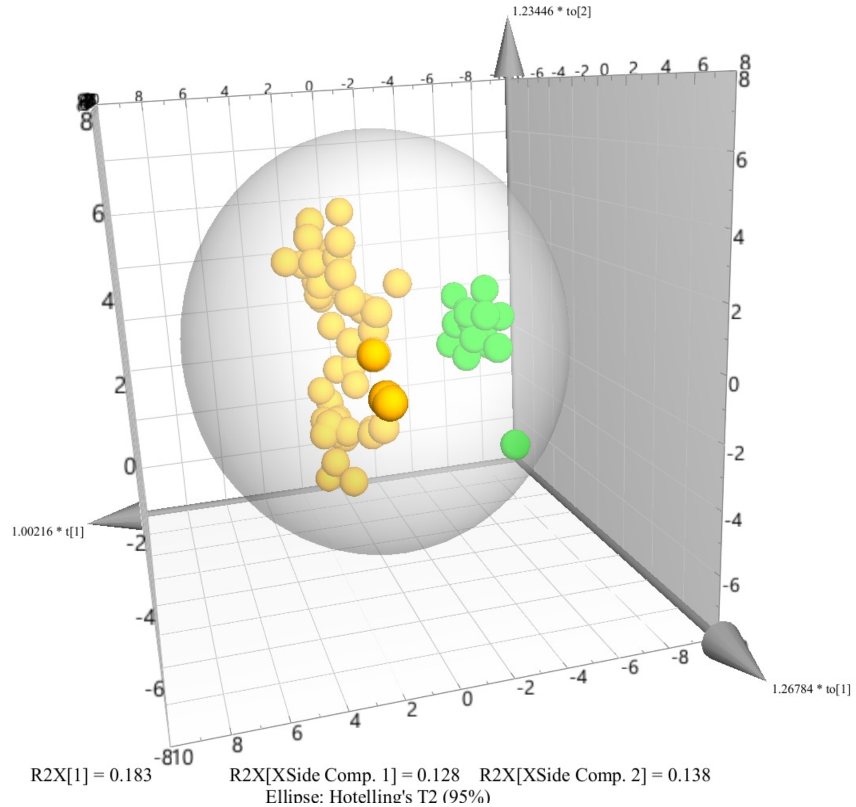

Figure S2. OPLS-DA score plot between acacia versus the rest

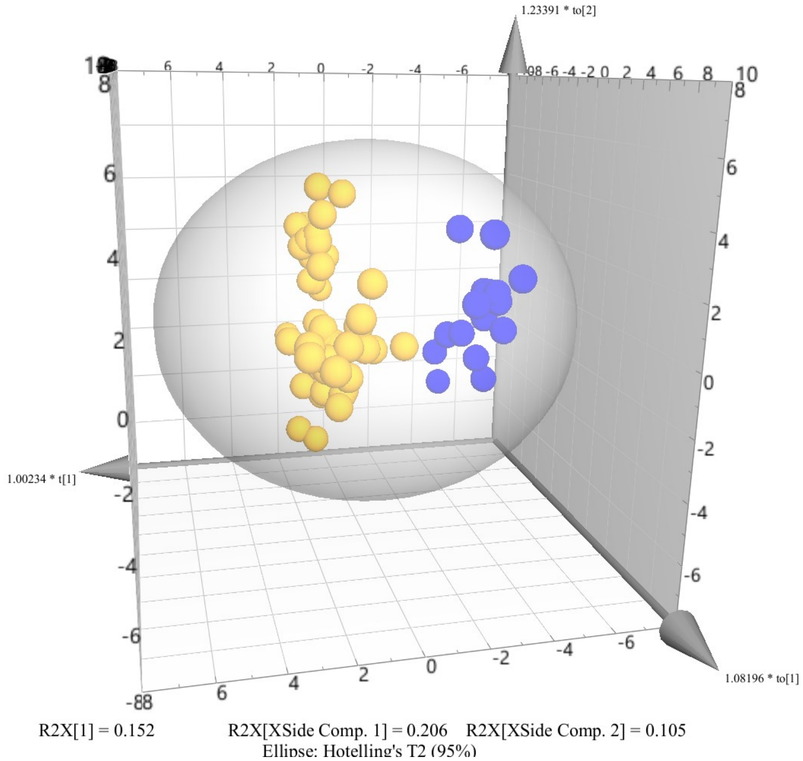

Figure S3. OPLS-DA score plot between vitex versus the rest

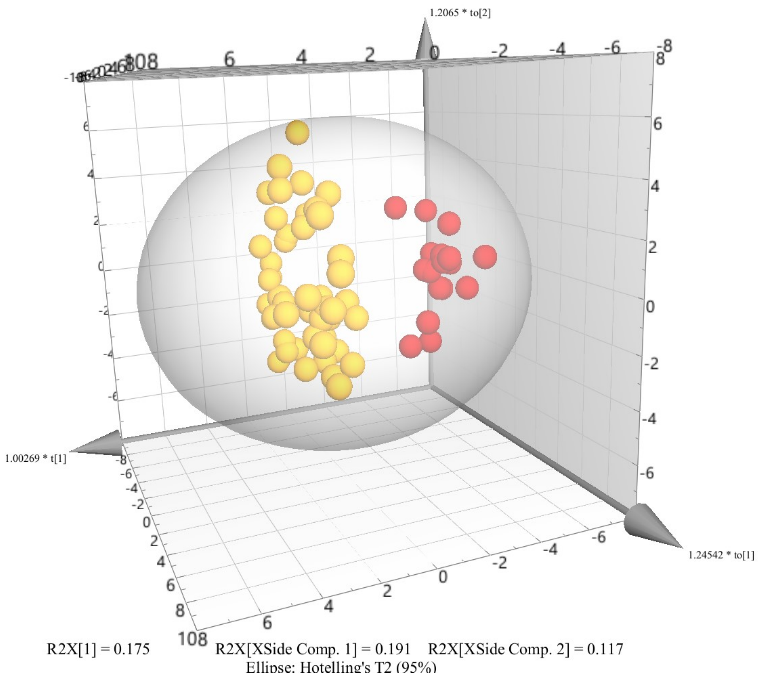

Figure S4. OPLS-DA score plot between linden versus the rest

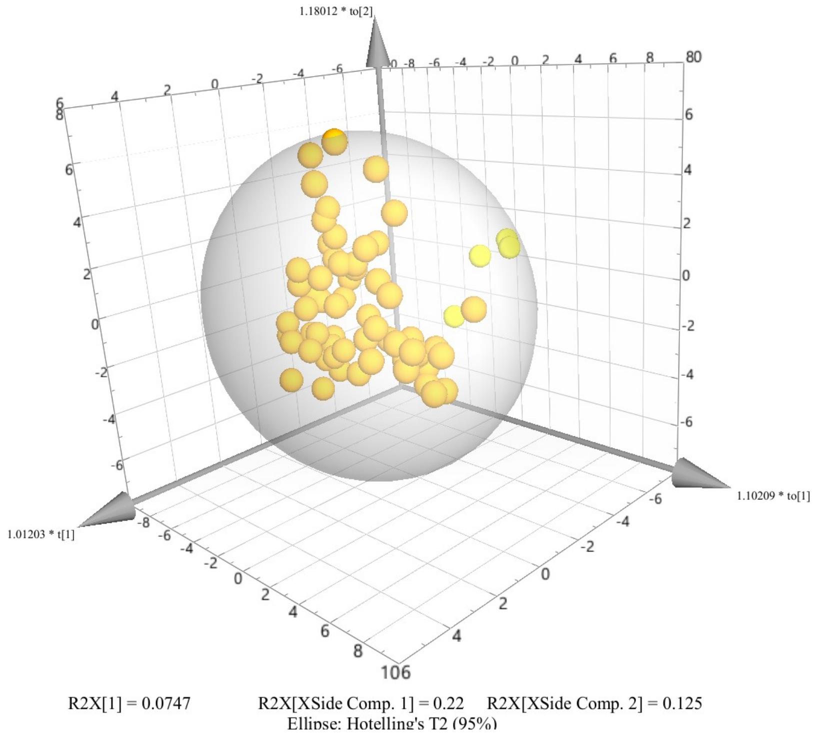

Figure S5. OPLS-DA score plot between rapeseed versus the rest

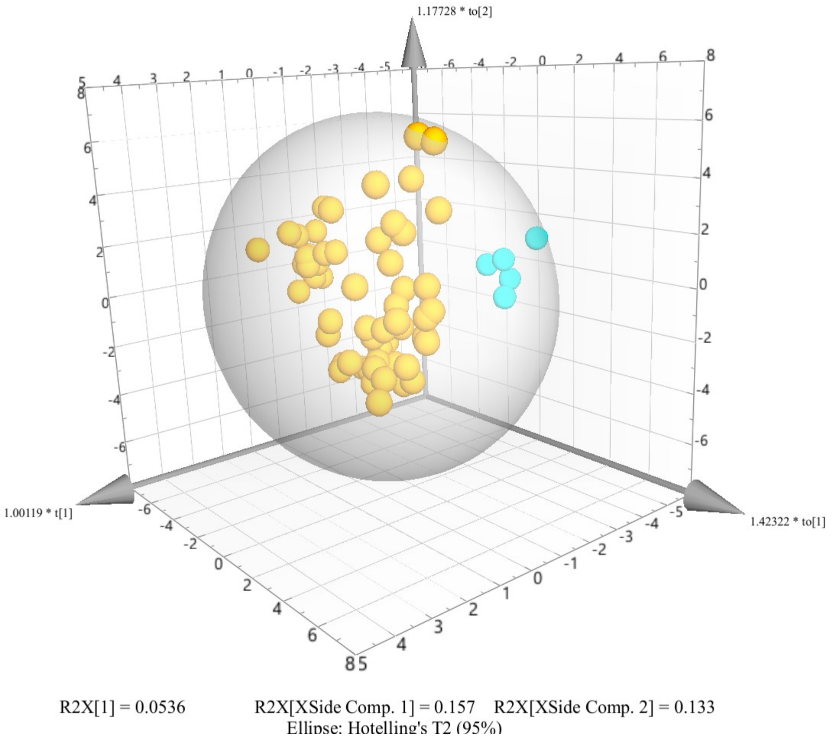

Figure S6 OPLS-DA score plot between *Astragalus* versus the rest

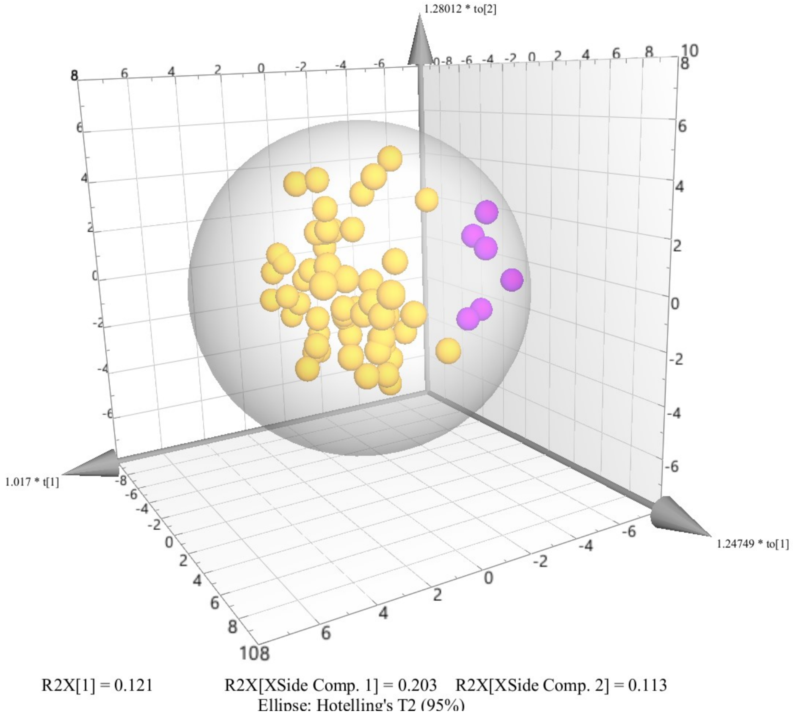

Figure S7. OPLS-DA score plot between *Codonopsis* versus the rest

Table S1 Content of phenolic and flavonoid compounds in acacia honey samples (ng g^{-1})

Compound	Ac1	Ac2	Ac3	Ac4	Ac5	Ac6	Ac7	Ac8	Ac9	Ac10	Ac11	Ac12	Ac13	Ac14	Ac15	Ac16	Ac17
Ferulic acid	53.21	70.26	38.88	55.94	46.57	50.84	43.56	33.56	54.89	44.58	30.98	36.79	33.87	51.26	12.08	25.99	30.59
Isoferulic	23.90	70.09	65.53	86.54	64.55	24.60	33.09	24.09	23.25	34.40	18.25	39.66	29.87	23.80	16.69	11.90	9.46
Syringic acid	22.06	11.82	28.05	11.24	11.44	7.21	8.85	12.16	5.63	4.21	12.88	10.03	4.00	3.38	8.67	5.75	4.34
Sinapic acid	9.08	6.29	7.25	7.06	15.30	5.41	5.03	2.91	3.72	4.55	2.72	1.42	2.41	2.45	1.13	2.07	1.78
Chrysin	5.56	9.86	20.04	16.69	13.70	43.26	90.80	139.81	35.26	39.13	80.66	133.30	25.02	22.83	40.35	26.35	16.28
Pinocembrin	18.14	16.38	16.04	21.93	15.66	16.89	52.86	98.67	37.60	26.15	5.19	43.60	27.03	33.30	3.05	18.40	1.98
Formononetin	0.25	0.40	0.53	1.18	1.40	5.69	6.55	3.27	3.59	3.89	3.77	2.69	1.51	1.20	ND	0.76	0.80
Apigenin	10.14	3.96	13.79	4.79	4.33	27.20	66.66	32.33	24.04	27.04	20.23	57.57	15.63	12.47	12.95	14.49	13.65
Genistein	0.14	0.46	0.03	0.54	0.28	1.10	1.24	0.80	1.33	1.42	1.72	0.35	5.15	4.77	0.07	1.77	0.89
Naringenin	7.44	17.59	54.27	45.37	16.53	8.64	24.21	34.19	11.12	41.88	13.35	35.79	10.73	21.90	15.63	51.91	8.64
Calycosin	0.53	0.66	0.93	0.63	1.29	2.53	1.40	1.66	1.26	1.48	1.53	1.03	1.48	1.03	ND	0.33	0.29
Luteolin	5.31	29.75	11.46	36.85	24.72	233.03	103.39	15.09	23.23	30.02	43.89	30.78	46.48	36.10	3.25	26.74	23.62
Kaempferol	10.51	121.89	168.75	77.62	73.18	260.24	309.99	214.55	268.27	220.40	128.26	556.02	156.67	224.74	32.67	106.33	36.01
Hesperetin	0.02	0.09	14.02	0.52	0.08	0.86	14.25	10.38	0.71	ND	ND	8.49	0.70	0.73	12.50	8.38	0.74
Quercetin	25.98	25.79	26.11	25.16	29.45	50.28	182.76	207.52	163.87	249.71	88.75	94.93	36.39	ND	21.58	59.16	28.59
(-)Epigallocatechin	67.17	84.93	65.43	44.79	120.32	6.64	69.56	88.43	67.96	44.98	122.15	38.83	94.74	72.92	5.46	34.32	81.32
Myricetin	ND	ND	ND	ND	ND	ND	ND	ND	ND	ND	ND	ND	ND	ND	ND	ND	ND
Ononin	0.19	0.03	0.41	0.16	0.26	0.24	0.98	0.45	0.09	0.07	0.31	0.42	0.02	ND	ND	0.02	ND
Genistin	0.11	0.16	0.18	0.16	0.46	0.30	0.32	0.36	0.23	0.22	0.25	ND	ND	ND	ND	ND	ND
Vitexin	0.78	1.38	2.94	1.33	2.03	3.46	5.93	1.33	1.17	1.05	1.86	1.33	1.60	1.10	ND	2.15	3.89
Calycosin-7-O-β-D-glucoside	0.04	0.03	ND	0.08	ND	ND	ND	ND	ND	ND	ND	ND	ND	ND	ND	ND	ND
Baicalin	0.37	0.37	0.39	0.44	0.48	0.74	0.63	0.58	0.44	0.47	0.57	0.57	0.44	0.39	0.52	0.40	0.41
4-Hydroxy-benzoic acid	511.15	315.10	450.69	296.89	696.02	371.48	539.37	528.89	394.20	306.50	436.20	424.17	261.38	287.20	162.31	372.72	483.40
3-Hydroxy-benzoic acid	<LOQ	<LOQ	<LOQ	<LOQ	<LOQ	<LOQ	<LOQ	<LOQ	<LOQ	<LOQ	<LOQ	<LOQ	<LOQ	<LOQ	<LOQ	<LOQ	<LOQ

Table S1. *Cont.*

Gallic acid	6.15	5.20	8.40	7.54	4.52	15.78	22.17	18.84	11.86	29.23	18.10	9.86	7.58	5.21	2.76	4.43	4.34
p-Coumaric acid	45.80	26.25	47.01	19.92	18.27	40.57	41.19	45.12	30.27	21.40	39.29	38.65	18.72	33.33	8.77	26.57	33.18
Salicylic acid	44.93	61.81	48.87	71.57	58.93	92.00	46.47	52.91	83.47	82.29	35.08	44.94	48.35	57.91	25.78	34.17	43.34
Caffeic acid	67.04	41.68	77.56	38.18	33.09	38.61	119.87	92.04	62.88	36.30	56.14	80.30	43.09	43.88	19.62	68.35	73.66
Abscisic acid	443.76	716.98	556.12	708.96	588.48	741.68	595.80	445.85	638.25	612.26	461.15	593.30	355.42	364.85	212.75	286.05	362.96
Isorhamnetin	7.54	7.88	8.36	8.19	7.90	23.51	52.56	101.83	77.23	70.25	23.80	33.63	22.43	<LOQ	5.58	41.36	16.98
3,4-Dihydroxy-benzoic acid	36.16	51.73	36.39	45.54	33.16	45.43	49.17	66.64	53.58	76.83	51.51	53.28	69.59	73.01	24.01	35.66	39.70
Chlorogenic acid	55.94	129.38	47.70	138.32	73.97	61.35	163.24	82.23	75.39	40.51	69.84	88.95	83.57	62.21	37.62	74.89	89.11
Rutin	9.02	3.23	9.24	5.53	1.93	6.24	5.56	5.27	5.94	6.32	2.51	4.04	1.88	1.44	1.50	4.06	2.17
Quercetrin	1.15	0.32	1.66	0.42	0.43	0.83	0.47	1.43	0.74	0.69	0.33	0.30	0.65	0.65	0.34	0.37	0.30
Hesperedin	1.44	0.03	2.30	1.36	3.29	75.95	45.74	33.18	40.06	32.34	51.82	0.64	0.93	1.05	0.76	1.01	0.85

Table S2. Content of phenolic and flavonoid compounds in *Vitex* honey samples (ng g^{-1})

**Compound**	**Vi1**	**Vi2**	**Vi3**	**Vi4**	**Vi5**	**Vi6**	**Vi7**	**Vi8**	**Vi9**	**Vi10**	**Vi11**	**Vi12**	**Vi13**	**Vi14**	**Vi15**	**Vi16**	**Vi17**
Ferulic acid	57.54	92.05	31.03	112.62	65.56	47.65	155.76	133.38	39.05	66.30	61.79	83.16	82.82	23.82	47.15	34.86	51.16
Isoferulic	98.43	15.19	19.84	17.76	68.06	84.38	37.97	121.31	27.76	32.18	36.73	42.83	50.04	50.37	20.52	43.00	31.50
Syringic acid	15.00	28.42	32.17	16.15	48.33	10.47	43.99	11.67	7.35	5.59	7.67	7.26	6.84	8.51	2.91	4.58	6.04
Sinapic acid	2.01	4.62	0.69	6.77	4.89	1.75	15.33	2.06	2.52	ND	1.83	ND	3.07	ND	4.49	2.36	2.78
Chrysin	64.03	51.12	23.32	38.71	106.60	14.98	13.30	111.87	79.01	72.74	99.15	242.66	173.56	32ND	13.77	114.92	82.93
Pinocembrin	153.26	115.80	20.59	71.74	54.92	34.51	59.55	174.32	88.60	52.91	78.20	175.25	160.05	80.12	51.08	156.20	162.04
Formononetin	ND	ND	ND	0.05	0.39	ND	ND	ND	ND	0.34	ND	0.21	0.25	0.01	ND	ND	0.11
Apigenin	193.38	155.14	56.67	123.39	161.37	78.20	134.97	181.10	80.27	150.10	215.86	358.91	302.00	303.23	77.34	282.08	131.67
Genistein	0.14	ND	1.59	ND	ND	ND	ND	0.68	5.43	4.92	2.55	6.92	ND	ND	1.42	ND	6.31
Naringenin	17.18	13.15	23.44	8.01	18.50	26.34	20.54	30.16	17.22	5.75	10.47	11.38	18.97	38.12	2.59	48.48	33.56
Calycosin	ND	ND	ND	ND	ND	ND	ND	ND	ND	ND	ND	ND	ND	ND	ND	ND	ND
Luteolin	187.80	162.57	56.70	152.83	179.10	103.26	129.61	169.90	151.21	83.15	141.96	230.34	165.35	268.95	59.23	156.82	87.06
Kaempferol	193.40	184.53	10.27	120.11	222.36	38.73	243.02	27.07	266.58	80.39	267.61	202.57	387.24	383.92	44.87	271.67	283.27
Hesperetin	7.05	2.12	7.05	2.12	7.05	2.12	7.05	2.12	ND	0.54	1.98	6.98	3.26	23.59	1.20	22.35	7.16
Quercetin	8.08	25.47	8.08	25.47	8.08	25.47	8.08	25.47	197.91	26.86	106.44	113.98	140.69	160.23	27.09	137.92	124.41
(-)-Epigallocatechin	14.94	21.29	ND	13.05	14.55	14.32	15.55	24.65	8.26	14.20	11.88	60.20	19.79	34.55	14.96	18.08	6.41
Myricetin	ND	ND	ND	ND	ND	ND	ND	ND	ND	ND	ND	ND	ND	ND	ND	ND	ND
Ononin	0.35	0.22	0.20	0.61	0.61	0.21	0.17	0.13	0.20	0.14	0.24	0.51	0.17	0.18	ND	0.10	0.11
Genistin	ND	0.07	0.24	0.23	0.36	0.07	0.16	0.12	0.17	ND	0.24	0.36	0.20	0.13	ND	0.06	0.02
Vitexin	51.47	44.71	32.88	40.33	59.26	59.53	36.46	46.41	46.07	42.08	34.70	68.01	53.49	87.29	19.81	46.62	29.28
Calycosin-7-O-β-D-glucoside	ND	ND	ND	ND	ND	ND	ND	ND	ND	ND	ND	ND	ND	ND	ND	ND	ND
Baicalin	0.41	0.52	0.39	0.64	0.39	0.44	0.39	0.48	0.56	ND	0.50	0.56	0.50	0.48	0.47	0.42	0.39
4-Hydroxy-benzoic acid	2818.85	2987.38	1229.77	2814.04	2464.38	2112.27	1981.94	1853.57	3051.94	2583.71	2584.91	3950.29	2634.45	4418.46	1724.60	2174.44	1850.33
3-Hydroxy-benzoic acid	13.71	10.27	<LOQ	11.03	17.39	7.68	4.63	1.25	<LOQ	<LOQ	<LOQ	<LOQ	<LOQ	18.76	<LOQ	11.91	<LOQ

Table S2. *Cont.*

Gallic acid	17.24	33.62	6.52	22.51	137.90	16.30	65.86	14.19	42.10	24.32	18.55	45.25	41.83	37.15	21.24	33.10	18.10
p-Coumaric acid	98.40	174.27	38.44	178.46	151.41	105.81	155.77	122.67	105.60	89.39	84.51	170.95	111.74	216.92	50.74	102.13	69.11
Salicylic acid	41.99	36.57	38.72	80.80	56.12	42.55	46.27	39.04	53.36	52.41	91.88	40.57	67.73	53.69	23.89	38.08	48.99
Caffeic acid	524.16	668.54	134.31	664.22	566.94	542.35	676.96	711.46	1058.85	877.19	784.95	1354.36	719.43	1758.10	449.80	962.72	679.88
Abscisic acid	97.41	139.67	68.38	108.05	425.41	105.64	746.47	152.26	205.84	135.89	205.86	131.85	242.76	143.73	65.09	149.41	150.51
Isorhamnetin	12.48	12.91	ND	14.10	11.86	9.57	32.26	46.20	41.40	10.58	34.89	23.13	35.52	28.46	9.98	28.78	24.03
3,4-Dihydroxy-benzoic acid	242.99	233.58	191.61	256.83	227.59	182.12	226.93	221.92	264.18	304.99	284.40	369.53	207.09	303.74	137.06	170.03	147.13
Chlorogenic acid	2787.84	2344.53	1597.33	4047.92	1398.61	4452.98	1734.03	4800.88	3543.39	3493.20	2497.68	6635.87	2330.26	5082.64	1458.99	3322.01	2386.46
Rutin	2.50	2.63	1.07	3.32	17.04	2.94	33.38	7.00	4.21	1.65	4.44	7.83	14.38	10.96	2.20	3.57	1.47
Quercetrin	2.13	4.36	0.38	1.49	52.03	1.37	15.31	0.85	0.62	0.39	0.56	1.01	1.24	1.66	0.40	0.64	0.46
Hesperedin	ND	4.36	ND	ND	ND	ND	ND	ND	0.74	0.04	2.97	2.46	1.88	5.89	1.76	1.18	0.62

Table S3. Content of phenolic and flavonoid compounds in linden honey samples (ng g^{-1})

Compound	Li1	Li2	Li3	Li4	Li5	Li6	Li7	Li8	Li9	Li10	Li11	Li12	Li13	Li14	Li15	Li16	Li17
Ferulic acid	47.78	134.72	127.42	86.77	141.90	79.58	9.47	108.06	44.59	49.62	122.58	50.61	169.16	50.56	50.74	30.59	53.61
Isoferulic	13.98	98.41	167.01	199.60	90.10	96.66	70.42	19.44	203.17	37.40	56.84	104.66	87.96	125.53	64.64	59.89	31.80
Syringic acid	16.35	9.71	7.61	6.86	4.43	8.19	38.62	3.82	11.22	9.18	10.23	11.51	12.86	9.85	7.89	8.09	8.39
Sinapic acid	ND	ND	ND	ND	ND	ND	ND	ND	ND	ND	ND	ND	ND	ND	ND	ND	ND
Chrysin	49.78	36.71	47.09	37.89	13.82	78.12	91.08	20.46	83.66	44.99	45.37	61.54	40.74	73.92	37.41	55.77	36.46
Pinocembrin	84.35	93.04	60.14	80.25	20.34	146.78	150.40	32.78	198.92	91.66	79.29	110.47	45.45	146.71	79.11	95.16	64.24
Formononetin	ND	0.14	0.23	ND	ND	ND	ND	1.04	ND	ND	ND	ND	0.14	1.49	ND	ND	ND
Apigenin	63.64	12.91	5.39	5.97	2.55	48.10	154.79	12.84	23.04	19.05	14.88	14.45	15.22	24.93	18.30	13.06	9.32
Genistein	0.72	1.12	0.27	ND	0.63	ND	1.12	ND	ND	ND	ND	ND	ND	ND	1.43	ND	ND
Naringenin	13.29	14.03	6.44	4.65	2.77	4.02	18.78	2.49	15.71	13.86	11.75	15.19	6.08	9.75	11.54	9.27	9.41
Calycosin	ND	ND	ND	ND	ND	ND	ND	ND	ND	ND	ND	ND	ND	ND	ND	ND	ND
Luteolin	24.01	18.69	17.20	23.82	3.02	7.38	45.97	11.94	27.62	14.32	10.56	15.03	25.59	13.35	2.95	10.19	8.60
Kaempferol	195.21	140.85	61.49	45.13	16.36	28.39	102.67	35.89	154.44	27.99	71.67	96.50	48.13	61.50	38.20	20.83	66.32
Hesperetin	7.63	23.66	2.06	11.12	ND	23.58	9.99	0.34	30.81	12.78	9.40	18.99	5.56	32.54	16.47	15.43	7.55
Quercetin	54.21	47.27	12.83	18.54	58.32	19.36	120.10	157.27	28.16	128.65	12.31	16.55	24.30	93.21	16.97	11.95	12.86
(-)-Epigallocatechin	68.62	25.46	140.87	101.25	109.97	91.00	143.54	268.22	91.17	26.95	ND	104.70	28.40	67.41	ND	ND	67.93
Myricetin	ND	8.97	5.95	3.68	4.01	ND	4.04	4.17	5.46	4.78	3.77	4.10	ND	3.76	4.02	4.92	7.13
Ononin	ND	0.04	ND	0.10	ND	0.04	0.07	ND	0.33	0.14	0.03	0.01	ND	ND	0.06	0.24	ND
Genistin	ND	ND	ND	ND	ND	ND	ND	ND	ND	ND	ND	ND	ND	ND	ND	ND	ND
Vitexin	14.66	ND	ND	0.74	0.47	ND	9.81	2.13	4.16	4.60	3.02	2.38	6.33	0.16	3.53	2.71	2.77
Calycosin-7-O-β-D-glucoside	ND	ND	ND	ND	ND	ND	ND	ND	ND	ND	ND	ND	ND	ND	ND	ND	ND
Baicalin	1.89	1.64	1.74	1.64	1.77	1.54	1.61	1.89	1.73	1.71	1.54	1.60	1.65	1.46	1.65	1.60	1.65
4-Hydroxy-benzoic acid	1079.21	460.38	505.27	498.69	393.79	565.46	886.21	356.80	658.42	556.32	526.97	604.04	452.63	506.03	664.76	623.58	459.78
3-Hydroxy-benzoic acid	<LOQ	<LOQ	<LOQ	<LOQ	<LOQ	<LOQ	<LOQ	<LOQ	<LOQ	<LOQ	<LOQ	<LOQ	<LOQ	<LOQ	<LOQ	<LOQ	<LOQ

Table S3. *Cont.*

Gallic acid	5.67	5.74	1.14	2.50	2.47	2.67	37.93	6.68	23.49	ND	ND	1.70	12.18	5.67	0.82	0.46	ND
p-coumaric acid	68.41	78.01	60.19	71.51	45.59	100.63	97.69	3.67	79.83	68.51	75.19	73.88	66.38	127.34	52.88	48.61	31.09
Salicylic acid	70.17	46.49	46.20	70.96	54.99	62.25	144.83	31.59	83.32	38.55	39.71	46.61	47.59	47.71	58.74	70.08	32.64
Caffeic acid	1116.66	419.68	177.88	170.82	170.34	369.24	579.54	29.06	581.78	114.55	219.00	299.95	577.62	476.28	283.82	75.34	91.14
Abscisic acid	235.04	483.40	770.10	566.01	426.18	372.69	302.71	9.54	484.35	301.08	305.78	352.30	323.30	486.46	245.41	246.87	170.87
Isorhamnetin	1.24	5.06	1.38	2.74	1.38	ND	1.96	2.95	6.80	3.16	3.35	2.65	2.62	1.59	2.00	2.42	2.11
3,4-dihydroxy benzoic acid	545.56	715.51	580.18	987.88	505.18	2424.19	554.14	177.86	1263.60	512.34	620.13	769.77	574.06	1453.39	906.20	923.74	464.01
Chlorogenic acid	58.59	36.06	ND	6.32	6.27	5.62	10.35	6.40	49.12	5.04	ND	ND	34.55	5.50	7.42	4.94	ND
Rutin	1.13	1.41	1.78	1.91	1.44	1.37	2.54	1.68	4.05	4.08	3.55	2.75	4.43	3.40	7.86	5.83	3.52
Quercetrin	0.82	0.77	0.66	1.25	0.68	12.85	1.19	0.42	2.07	0.81	0.78	1.48	1.41	2.93	1.38	1.34	0.63
Hesperedin	0.52	0.93	0.62	ND	0.86	0.33	1.37	1.23	1.89	ND	0.57	1.10	ND	0.64	1.34	1.17	ND

Table S4. Content of phenolic and flavonoid compounds in rapeseed, *Astragalus* and *Codonopsis* honey samples (ng g^{-1})

**Compound**	**Ra1**	**Ra2**	**Ra3**	**Ra4**	**As1**	**As2**	**As3**	**As4**	**As5**	**Co1**	**Co2**	**Co3**	**Co4**	**Co5**	**Co6**
Ferulic acid	14.59	16.19	18.13	47.05	17.52	19.19	26.35	42.68	31.89	21.57	65.74	20.63	36.93	23.35	64.05
Isoferulic	12.33	13.03	17.26	38.53	20.95	5.94	26.97	41.18	41.83	4.79	21.90	32.57	39.72	32.37	23.18
Syringic acid	61.13	46.75	140.82	44.48	5.04	8.68	12.52	19.36	10.97	25.52	43.51	17.51	19.36	14.08	23.22
Sinapic acid	2.41	2.95	6.37	7.94	2.85	0.69	2.05	2.05	2.21	1.95	5.33	1.02	2.00	1.44	3.91
Chrysin	43.51	55.75	53.57	107.39	69.51	65.58	72.43	62.97	133.11	110.12	82.76	99.43	101.26	73.06	245.76
Pinocembrin	33.78	6.61	8.69	86.46	53.36	74.91	53.51	41.10	94.04	75.97	45.89	87.55	80.79	59.46	128.01
Formononetin	ND	ND	ND	ND	18.59	14.02	5.79	5.35	19.52	8.58	19.52	4.27	3.15	10.05	7.63
Apigenin	6.54	14.83	70.47	10.69	28.70	887.47	37.06	145.22	34.53	301.82	208.91	403.28	190.27	174.00	282.18
Genistein	ND	ND	ND	ND	6.27	1.61	5.71	1.24	4.13	3.06	7.46	ND	0.54	2.68	ND
Naringenin	17.05	26.43	11.64	31.58	16.76	25.16	42.40	12.92	55.47	16.90	14.17	20.21	13.51	17.50	16.33
Calycosin	ND	ND	ND	ND	41.25	45.95	10.19	6.58	15.02	13.73	15.02	2.43	0.90	20.11	2.83
Luteolin	3.97	5.09	3.00	3.11	62.90	65.56	43.47	87.23	46.70	39.40	104.08	41.31	29.19	42.70	20.58
Kaempferol	640.10	574.48	504.93	941.61	513.53	682.41	348.12	1570.31	263.00	755.70	1867.88	319.33	129.15	548.13	207.30
Hesperetin	0.02	0.19	0.03	0.87	1.85	4.28	19.32	2.25	14.80	3.55	5.64	14.71	4.48	3.66	3.98
Quercetin	340.20	232.86	874.94	ND	647.67	1054.61	334.51	15.91	153.72	534.60	462.40	354.26	206.30	458.90	66.39
(-)-Epigallocatechin	ND	ND	ND	ND	ND	ND	ND	ND	ND	ND	ND	ND	21.59	23.33	25.69
Myricetin	5.80	5.55	5.07	3.21	9.43	12.84	7.52	4.10	3.70	20.95	19.01	14.65	8.06	12.58	9.10
Ononin	ND	0.07	0.06	0.44	0.33	0.43	0.36	0.29	0.32	0.46	0.43	0.63	0.51	0.44	0.77
Genistin	ND	ND	ND	ND	ND	ND	ND	ND	ND	ND	ND	ND	ND	ND	ND
Vitexin	1.36	7.44	ND	ND	1.59	1.31	6.36	3.39	3.08	3.09	18.67	ND	ND	2.55	3.51
Calycosin-7-O-β-D-glucoside	ND	ND	ND	ND	ND	ND	1.54	ND	ND	ND	ND	ND	ND	0.21	ND
Baicalin	0.94	0.93	0.90	0.91	0.92	0.90	0.97	0.92	0.94	0.83	0.94	0.87	0.87	0.80	0.77
4-Hydroxy-benzoic acid	774.67	1005.78	2255.48	808.39	988.20	925.10	985.78	797.36	620.75	895.61	1159.95	852.58	950.78	652.33	1034.08
3-Hydroxy-benzoic acid	<LOQ	<LOQ	<LOQ	<LOQ	<LOQ	<LOQ	<LOQ	<LOQ	<LOQ	<LOQ	<LOQ	<LOQ	<LOQ	<LOQ	<LOQ
Gallic acid	2.41	41.25	4.36	8.89	19.00	18.89	39.33	100.55	25.34	58.78	214.12	45.21	124.84	94.40	133.72

Table S4. *Cont.*

p-Coumaric acid	57.88	108.99	57.39	206.72	33.33	61.68	64.06	112.58	62.86	87.16	126.90	110.53	89.36	55.37	199.70
Salicylic acid	177.00	55.56	82.95	52.12	94.72	84.26	80.84	118.07	53.31	115.77	88.97	116.31	92.35	104.91	52.14
Caffeic acid	13.00	18.46	6.52	56.54	60.78	109.79	121.29	105.04	142.53	91.81	183.09	124.82	133.34	56.49	407.22
Abscisic acid	114.76	88.00	88.91	348.49	102.48	90.34	313.05	275.45	487.75	606.92	690.30	397.61	269.21	348.20	246.66
Isorhamnetin	28.88	31.24	92.97	66.47	197.58	229.85	105.01	20.15	69.69	115.84	107.75	111.56	80.99	94.19	35.63
3,4-Dihydroxybenzoic acid	218.47	145.63	176.61	61.37	185.77	106.63	132.39	148.16	60.90	307.88	195.87	444.16	686.77	433.21	155.20
Chlorogenic acid	ND	5.05	5.18	5.19	35.35	ND	156.86	ND	144.67	ND	224.27	28.63	153.20	22.11	31.14
Rutin	0.46	0.76	ND	2.54	1.37	7.64	5.07	ND	5.77	10.77	10.40	53.58	24.63	8.54	27.79
Quercetrin	0.33	0.32	0.25	3.04	0.63	3.40	3.00	1.18	2.65	6.98	3.98	11.56	5.57	2.47	1.44
Hesperedin	ND	ND	1.91	1.88	1.44	ND	17.11	8.43	4.26	22.15	0.57	ND	ND	5.62	ND
